# Exploration during early life: distribution, habitat and orientation preferences in juvenile king penguins

**DOI:** 10.1186/s40462-019-0175-3

**Published:** 2019-10-21

**Authors:** F. Orgeret, C. Péron, M. R. Enstipp, K. Delord, H. Weimerskirch, C. A. Bost

**Affiliations:** 1 Centre d’Etudes Biologiques de Chizé, CNRS, UMR 7372, 79360 Villiers en Bois, France; 2 Laboratoire de Biologie des Organismes et Ecosystèmes Aquatiques (BOREA), MNHN, CNRS, IRD, SU, UCN, UA. CP 26, 43 rue Cuvier, 75231 Paris Cedex 05, France; 3 Département Ecologie, Physiologie et Ethologie, Université de Strasbourg, CNRS, IPHC, UMR 7178, F-67000 Strasbourg, France

**Keywords:** King penguins, First year at-sea, Juveniles, Distribution, Ocean current, Wind, Habitat preferences, Cluster analysis, Net squared displacements, Orientation, Seabird, Foraging

## Abstract

**Background:**

The early life of marine apex predators is poorly known, particularly for diving species. The orientation and foraging skills are presumably less developed in juveniles than in adults, especially during their first year at sea when juveniles might disperse further than adults.

**Methods:**

Over two years of monitoring, we tracked the movements of 17 juvenile king penguins (*Aptenodytes patagonicus,* ~ 1 year old) using satellite relay tags from Crozet Archipelago (Southern Indian Ocean), starting when birds left their natal colony for the first time. For comparison we also tagged 6 non-breeding adults, which at that stage, similar to juveniles, are unhampered by reproductive constraints and might roam further than breeders. We used a combination of cluster analysis and habitat modelling to investigate and compare the movement patterns and habitat use of experienced (non-breeding adults) and non-experienced (juveniles) individuals.

**Results:**

While juvenile penguins and non-breeding adults followed similar routes, the movements by adults started later in the season and ranged over a considerably smaller area than juveniles. Net squared displacement analysis revealed that both groups did not move to a specific wintering area. Changes in direction of juveniles in respect to their departure island were similar and synchronous for both years. Habitat models revealed that foraging behaviour was affected by environmental variables such as wind or current speeds, sea surface temperature, or oceanic productivity, for both stages. Analysis of tracks revealed that birds moved predominately perpendicular or against the main direction of the Antarctic Circumpolar Current and the prevailing wind during austral summer (juveniles only) and autumn (juveniles and non-breeding adults). However, both juveniles and adults were more likely to move against the prevailing winds if productivity increased along their trajectories.

**Conclusions:**

The exceptional duration of our tracking study provided unprecedented insights into the distribution, habitat preferences and orientation of two poorly known life history stages of an expert avian diver. Our study suggests that juveniles might use both innate and learnt skills to reach profitable foraging areas during their first year at sea, which is critical in long-lived species.

## Background

In vertebrates, the period following emancipation of juveniles from their parents is critical [1]. During their first years, juveniles have, in general, a lower survival rate than adults [2]. The higher mortality of juveniles might be largely explained by a lack of proficiency concerning various behavioral aspects, such as foraging skills, predator avoidance and/or social interactions [3]. Many studies have shown that in long-lived species the foraging skills of juveniles are less proficient than those of adults [3]. Such differences are mostly related to physiological and developmental constraints [4], and the time needed to obtain sufficient knowledge about food acquisition and the environment to improve foraging skills [5]. Furthermore, the improvement of skills is strongly dependent on the particular environment encountered and various temporal and/or spatial scales [6].

The first foraging behaviour of many juvenile animals is presumably influenced by a combination of innate processes and acquired skills, with juveniles either following adults, or dispersing independently [7]. However, for many species the processes that shape both the ontogenetic changes in movement behavior and the choice of foraging destination remains unknown and are still key questions in movement ecology [8–11].

Locomotion skills and the capacity to benefit from environmental cues are less developed in juveniles, when compared with adult organisms [3]. Accordingly, juveniles are presumably less efficient at finding profitable food patches than adults [12]. Furthermore, during their first days of independence, juveniles depend largely on their innate skills with respect to orientation capacity (recognizing and maintaining direction). In several vertebrate taxa, the poor navigational skills of juveniles during their first months following their emancipation, is mostly associated with exploratory behaviour e.g. [12–14].

Juvenile seabirds often disperse over large oceanic areas and for extended periods, covering considerable distances (e.g. in procellariforms, > 1000 km and for several years, [14]), which makes investigation difficult. Thus, previous studies investigating the distribution of juvenile seabirds have largely relied on ship-based observations. However, due to the diving lifestyle of penguins, it is difficult to obtain reliable ship-based observations to investigate the distribution of both adult and juvenile penguins.

Biologging has revolutionized the study of penguin distributions and movements e.g. [15] and remains the best method to study their at-sea behaviour. Nevertheless, tracking penguins at sea remains challenging, as electronic tags attached to their back feathers can alter their diving behaviour by adding hydrodynamic drag [16, 17]. Most tracking studies have focused on breeding adults due to the increased possibility of recovering electronic tags when they return to land to care for offspring. In contrast, tag recovery is difficult or even impossible for juvenile and non-breeding penguins since they often disperse over vast ocean sectors for multiple months or years before returning to their natal or breeding colony [18–21].

Penguins are climate change sentinels and represent the largest seabird biomass in the Southern Ocean [22]. Changes in resource accessibility and acquisition are suspected as drivers of observed trends in breeding success and survival rate of penguins [15, 23, 24]. Hence, it is of critical importance to study the at-sea distribution and foraging behaviour of penguins during different life-history stages to better understand how it may influence their demographic parameters. Furthermore, few studies have investigated the diving behavior of juvenile penguins, a critical period of their life [18, 21, 25–27] with only two studies linking such an investigation with spatial distribution patterns of birds [18, 27].

In a pioneering study, Pütz and colleagues investigated the distribution of juvenile king penguins (*Aptenodytes patagonicus* Miller) from South Georgia and the Falkland islands [19]. They showed that juveniles traveled within a large area, moving between the Pacific and Atlantic Oceans and into the Indian Ocean. Environmental variables, such as Chlorophyll a, sea surface temperature and bathymetry were good predictors of the penguins’ distribution. The authors concluded that inexperienced penguins developed their foraging skills over time and speculated that ocean currents might play an important role for their orientation [19].

Here, we investigated both the at-sea distribution and the diving behavior of juvenile king penguins during their first year at sea, using small and streamlined satellite relay archival tags. Our aim was to investigate the dispersion of juvenile king penguins from the Crozet Archipelago, Southern Indian Ocean. We compared the unexperienced at sea behavior of juveniles to that of experienced non-breeding adults, which, similar to juveniles, are unburdened by reproductive constraints. In particular, we investigated:

1. the movement patterns of juvenile and non-breeding adult king penguins and whether they move to a specific wintering area before returning to the colony;
2. the environmental variables determining the foraging behavior of juveniles and non-breeding adults, and the potential ability of juveniles to detect/follow environmental cues;
3. juvenile orientation preferences and the possible link between movement trajectories and prevailing ocean current and wind directions as potential information sources for ocean productivity.

## Methods

### Study site and breeding cycle of king penguins

Situated in the Southern Indian Ocean, the Crozet Archipelago (46.43°S, 51.86°E) hosts one of the largest king penguin populations in the world [28, 29]. These birds are long-lived (average lifespan: ~ 20 years) and sexually mature between 3 and 4 years old. Females lay a single egg between November and February with juveniles fledging the following November–March [28]. Upon fledging (~ 1 year old), juveniles leave their natal colony for the first time and spend one year at sea before returning to land for their first annual moult [30]. The majority of king penguins breed annually however a small proportion of birds (~ 13% per year [31]) irregularly skip a reproductive season and during this time, are considered as ‘non-breeders’.

### Logger settings and deployments

Field work was conducted at the “La Grande Manchotière” colony (20,000 breeding pairs) on Possession Island, Crozet Archipelago, during 2013/2014 and 2014/2015. Tracking data were collected from juveniles (*N* = 17) and non-breeding adults (*N* = 6). Almost all juveniles left the colony between the end of November and the end of December (the beginning of the austral summer) whereas, non-breeders left the colony between mid-February and mid-March, after completion of their annual moult. All birds were equipped with SPLASH tags (SPLASH10–283; Wildlife Computers, Redmond, WA, USA) just before their departure to sea.

Tags (109x32x26 mm; LxWxH; mass: 62 g, ~ 0.3% of a bird’s body mass) were fixed to the feathers of the middle lower back using Loctite glue and cable ties. The tags had a flexible antenna which was 8 cm long, 1.6 mm in diameter and faced backwards at an angle of ~ 45°. SPLASH tags are composed of a logger module, which collects and processes pressure data and a transmitter module (Argos PTT) which relays data summaries via Argos satellites and enables the computation of locations by the Argos system. Due to battery limitations, tags were programmed to record and transmit dive data every 3rd day (i.e. 1 day ON, 2 days OFF), while locations were computed on average every two hours. During a recording day, pressure was sampled continuously at a rate of 1 Hz and was processed by an on-board algorithm to create a dive-profile log. During processing, depth data were divided into ‘dive’ and ‘surface’ events, from which dive profiles were reconstructed which included information on maximum dive depths, dive durations, and time spent at the surface between dives. The dive-profile log was transmitted via Argos satellites when birds returned to the surface. Due to failed transmission attempts, an average (±SD) of 55.0 *±* 13.1 dives (> 2 m) were relayed per recording day (i.e. every 3rd day), while an average (±SD) of 14.5 *±* 1.6 locations were computed for each day.

### Environmental variables

Environmental variables were either extracted directly from online data portals or estimated from indirect oceanographic data products (e.g. oceanic fronts).

Sea Surface Temperature (SST, in °C), Mixed Layer Depth (MLD, in meter), Sea Ice Concentration (SIC, in percentage), Chlorophyll a concentration (CHLA, in mg/m^3^) and Total Oceanic Current speed (in m.s^− 1^) and direction were downloaded from the Copernicus Marine Environment Monitoring Service of the European Union (http://marine.copernicus.eu/; Global Ocean Physics Analysis and Forecast), with a spatial resolution of 1/12° at a daily temporal resolution. The ocean circulation data provided by this service are based on the NEMO model (Nucleus of European Modelling of the Ocean [32]) and includes all current components. As CHLA satellite measurements are sparse for the Southern Ocean, especially during winter, when extended cloud coverage prevents measurements [33], the model outputs extracted from the *Global Ocean Biogeochemistry Analysis and Forecast* database with a spatial resolution of 1/2° at a weekly temporal resolution were therefore used. Wind speed (in m.s^− 1^) and direction data were downloaded from the Global Forecast System of the United States National Weather Service with a spatial resolution of 1/2° degrees at a temporal resolution of 3 h (https://www.ncdc.noaa.gov/).

**Fig. 1:**
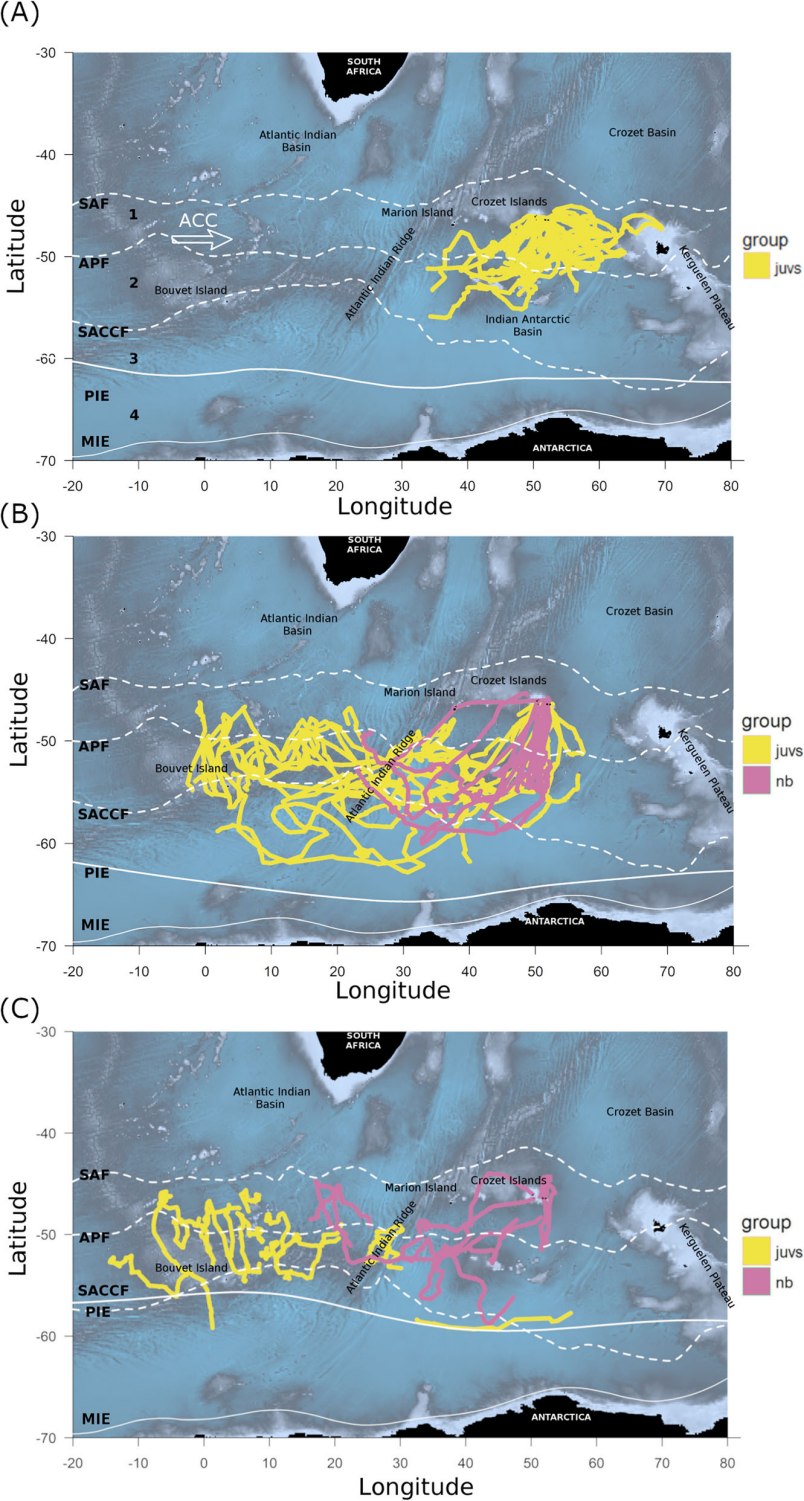
Argos tracks of juvenile (juvs, N = 17), and adult non-breeding king penguins (nb, N = 6) from Crozet Archipelago across seasons (**a**: Summer, **b**: Autumn, **c**: Winter; data for both years were pooled). The seasonal mean positions of the major oceanic fronts within the area are indicated by dashed lines: the Sub-Antarctic Front (SAF), the Antarctic Polar Front (APF) and the Southern Antarctic Circumpolar Front (SACCF). Solid white lines represent the mean positions of the northern limits for the Pack Ice Extent (PIE) and the Minimal Ice Extent (MIE) in February, as indicated. Numbers in (**a**) indicate the different zones delimited by oceanic fronts 1: Polar Frontal Zone (PFZ), 2: Antarctic Zone (AZ), 3: Southern Antarctic Circumpolar Zone (SACZ), 4: Seasonal Pack-Ice Zone (SPIZ). Main direction of the Antarctic Circumpolar Current (ACC) is also indicated

The location of oceanic fronts (Fig. 1) was estimated using Sea Surface Height (SSH) values following [34]. SSH data were downloaded from Aviso (http://www.aviso.altimetry.fr) with a spatial resolution of 1/4° at a daily temporal resolution. In Fig. 1, SSH data were averaged for both years during which birds were tracked.

### Data formatting, analysis and statistics

Data were formatted and analysed using R software, R3.2.2 (R Development Core Team 2016). All values are presented as means +/− standard deviation, unless stated otherwise.

Given the relatively small sample size, data from both years were pooled in the analysis, unless stated otherwise. All graphs were plotted with the *ggplot2* package [35].

#### Argos locations

Erroneous Argos locations were filtered using the speed filter within the R package *argosfilter* [36], applied to the travel speed. The maximum travel speed was fixed to 14 km h^− 1^ [37]. The speed filter procedure removed 15% (*n* = 4283) of all received Argos locations (*n* = 28,449). The average speed of the Antarctic Circumpolar Current (ACC) was low (0.11 ± 0.11 m.s^− 1^) in comparison to the travel speed of both juveniles and non-breeding adults (~ 1.1 ± 0.2 m.s^− 1^). The effects of the currents on the trajectories of the individuals were thus negligible, and the current corrected movements were therefore not implemented in further analyses [38].

#### Movement patterns

To investigate whether juveniles and non-breeding adults occupy a specific wintering area, Net Squared Displacement (NSD) analysis and a latent state model, within the *lsmnsd* package [39], was first used. NSD analysis measures the squared distance (great circle distance calculated with the *argosfilter* package) between each average daily track location of an individual and the first recorded location of its trip. The latent state model then distinguishes between three states from the NSD distribution: ‘state 1’ (in summer) and ‘state 2’ (in winter) correspond to different geographical areas where movements were concentrated (equivalent to two encamped movement modes), while ‘state 3’ corresponds to movements outside and between these areas (equivalent to a transiting mode). These states are used to describe different phases along the trajectories of the birds. Then using ‘state 2’, as classified by the latent state models, their winter range was identified. To test if individuals targeted specific environmental features during winter (i.e. if they remained in an area with favourable conditions), a Principal Component Analysis (PCA) was then used to assess the inter- and intra-individual variability of environmental variables associated with daily locations during ‘state 2’.

#### Inference of behavioral modes (traveling versus foraging dives)

To distinguish between traveling and foraging dives within the movement trajectories of birds, a cluster analysis was used (following [40]) based on both traveling speed and dive parameters (dive duration, maximum depth, and surface interval duration). Travel speed was calculated by dividing the distance between two consecutive dive locations (using the great circle distance with the *argosfilter* package) by the time difference between the initiation of the respective dives (i.e. including dive duration). We then computed hourly averages of the traveling speed and dive parameters.

A distance matrix was then calculated to determine the similarity between these hourly averaged dive parameters and traveling speeds. A ‘Manhattan’ measures was used to estimate the distance matrix and created a hierarchical cluster tree, using unweighting average distance with the *HCPC* function in the *FactomineR* package [41]. A cluster analysis was run separately for juveniles and for non-breeding adults. Based on resulting clusters (see Results), daily dive locations were classified as two different dive modes: ‘traveling’ or ‘foraging’. The tag of one non-breeding adult failed to record dive data and was, therefore, not included in this analysis.

#### Habitat preferences (environmental features)

To better understand the environmental features that juveniles and adult non-breeders associated with different dive modes, the binary outputs of the cluster analysis (traveling = 0; foraging = 1) were modeled as a function of environmental variables (extracted for the associated locations of birds, using the *SDMTools* packages, [42]). For this, generalized additive mixed-effects models (GAMMs; *mgcv* package) [43] were used. GAMMs were used because they allow for non-linear relationships between response variables and environmental covariates, which is typical in habitat studies [44]. A two-dimensional spline was implemented on geographical coordinates projected into Lambert Equal-Area Azimuthal to implicitly include some spatial structure of the data [45]. To account for the hierarchical structure of our data, individual identification was included as a random effect in the models. Year, stage (juvenile versus non-breeder) and season (summer, autumn, winter) were integrated as categorical variables in the fixed effects part of each model. GAMMs were fitted with a binomial error distribution and a *logit* link function. To avoid over-parameterization, all environmental covariates were smoothed by fitting cubic regression splines with shrinkage [46].

Model selection was conducted using Akaike’s Information Criterion (AIC) values to rank all possible model combinations according by their degree of parsimony (*dredge* function of the MuMIn package) [47]. Restricted maximum likelihood was used for model estimation and the model with the lowest AIC value and a ΔAIC> 2 (compared with other candidate models) was retained [48]. Fitted values were back-transformed to probability, using the *plogis* function. Prediction maps for foraging probability were plotted using the *vis.gam* function and also show back-transformed estimates.

#### Orientation preferences

The penguins’ orientation preferences related to the oceanic current and wind were investigated by calculating the angular difference (*circular* package [49]) between animal heading and current and wind directions, respectively. Since there was a mismatch between the number of transmitted Argos locations per day and the available temporal resolution for current and wind datasets, Argos locations were re-interpolated at 12 h intervals (i.e. 2 locations per day). The *zoo* package with the *na.approx* function [50] was used as this function was specifically designed for irregular time series. The angular difference between the observed animal track (‘heading’ = 0/360°) and the current or wind directions (separately) was calculated. Angular difference values between 315° and 45° indicate a similar direction for both bird trajectory and current or wind (downstream orientation). By contrast, values between 135° and 225° indicate an opposing direction for bird trajectory and current or wind (upstream orientation, see [37, 38]). All remaining values indicate a perpendicular direction.

Differences in the proportion of downstream and upstream orientation of the birds were assessed with Generalized Linear Mixed Models (GLMMs, lme4, [51]), with the proportion of downstream versus upstream orientation used as the response variable (for current and wind separately). A binomial error distribution was used and individual identification was included as a random effect within models. Post-hoc pairwise comparisons between stage and season were made with the *lsmeans* package [52], using the Bonferroni procedure [53].

Additionally, the effect of ocean productivity on bird orientation with respect to prevailing current and wind directions (upstream/downstream) was investigated. The binary outputs of bird orientation (downstream = 0, upstream = 1) was modelled as a function of the CHLA concentration (used as a proxy for ocean productivity) at location at time (t) and the following location at time (t + 1) along the trajectories (at 12 h intervals). Given the potential non-linearity of the relationship, GAMMs were used with a binomial error distribution and a *logit* link function. Models were run separately for wind and current directions, while stage (juvenile versus non-breeder) and season were integrated as categorical variables in the fixed effects part of each model and individual identification was included as a random effect.

## Results

### Tracking duration and distances traveled

Juveniles and non-breeding adults were tracked for an average duration of 206 ± 61 days and 220 ± 73 days, respectively (Additional file 1: Tables S1&S2). The total cumulative distance traveled by juveniles ranged between 3317 and 13,259 km, while non-breeders traveled between 8487 and 11,664 km (Additional file 1: Tables S1&S2). The distance between furthest location (maximal distance) from the natal colony ranged between 1004 and 4655 km (up to 14.3°W and 62.0°S) for juveniles. In comparison, maximal distance for non-breeders ranged between 1461 and 2693 km, respectively. See Additional file 1: Tables S1&S2 for individual tracking parameters. Five individuals had shorter monitoring durations (3 to 4 months) compared to the others (Additional file 1: Table S1).

### Seasonal distribution at sea

Juveniles and non-breeding adults divided their time equally between the Polar Frontal Zone (PFZ) and the Antarctic Zone (AZ) resulting in a similar latitudinal distribution (north-south) of juveniles and non-breeders during both years (Fig. 1 & Additional file 1: Figure S1). Interestingly, the Sub-Antarctic Front (SAF) was the northern distribution limit for both juveniles and adult non-breeders. However, juveniles moved much further west than non-breeders did (by more than 2000 km). Three juvenile birds moved far south and came close to the ice edge (< 150 km from the ice edge, Fig. 1), with one juvenile reaching the ice edge (up to 27% of sea ice concentration). Non-breeders did not travel as far south, with their minimum distance to the ice edge being ~ 250 km.

### Movement patterns

**Fig. 2:**
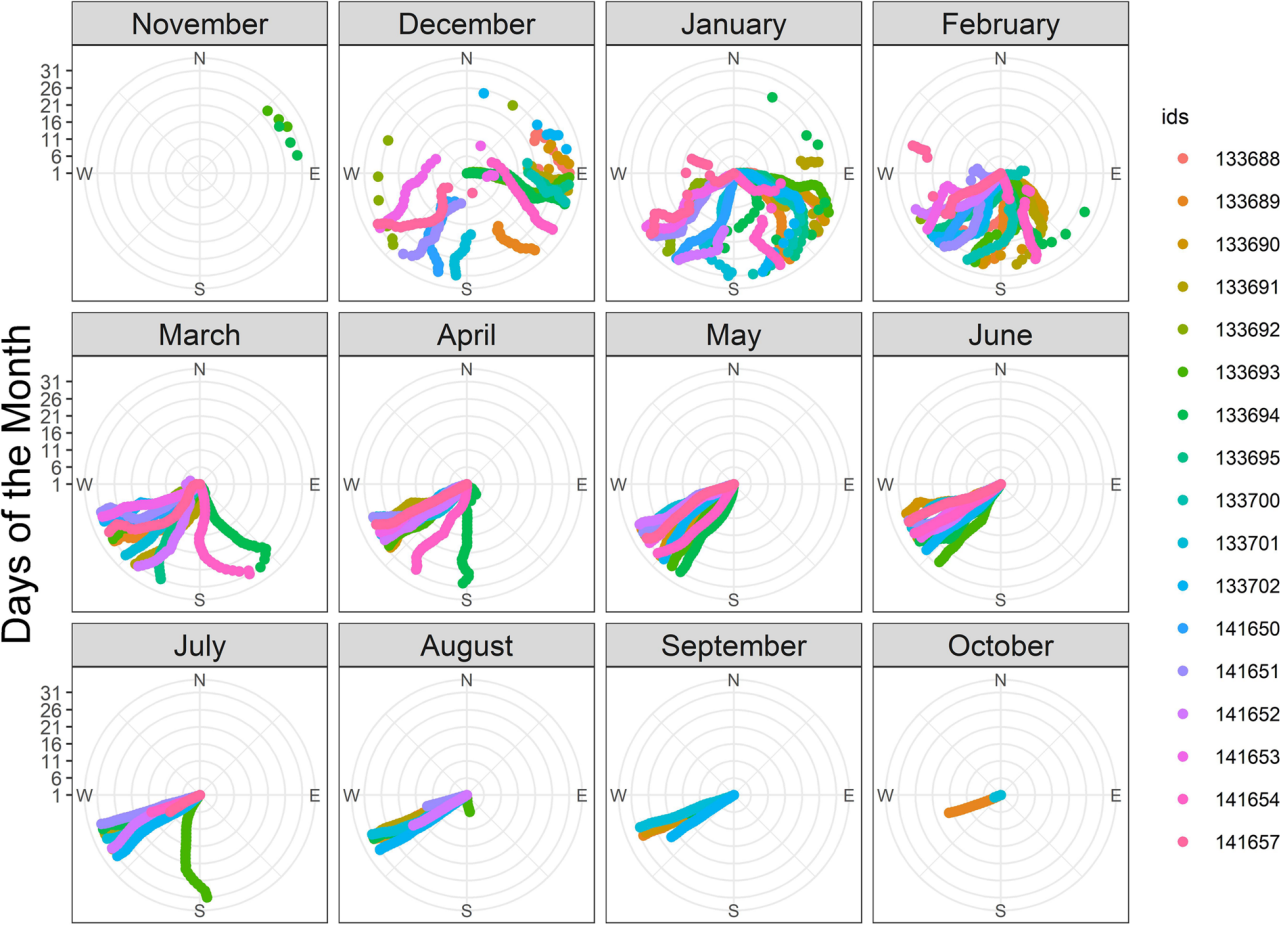
Daily directional changes of 17 juvenile king penguins (colour-coded) relative to their departure colony (innermost circle). Daily averages compass bearings (*circular* package [49]) are presented for each month, starting with juvenile departure in November until October of the following year. Within a monthly circle, time progresses from the inside out, with the outermost ring representing the last day of the month. Data for both years were pooled

**Fig. 3:**
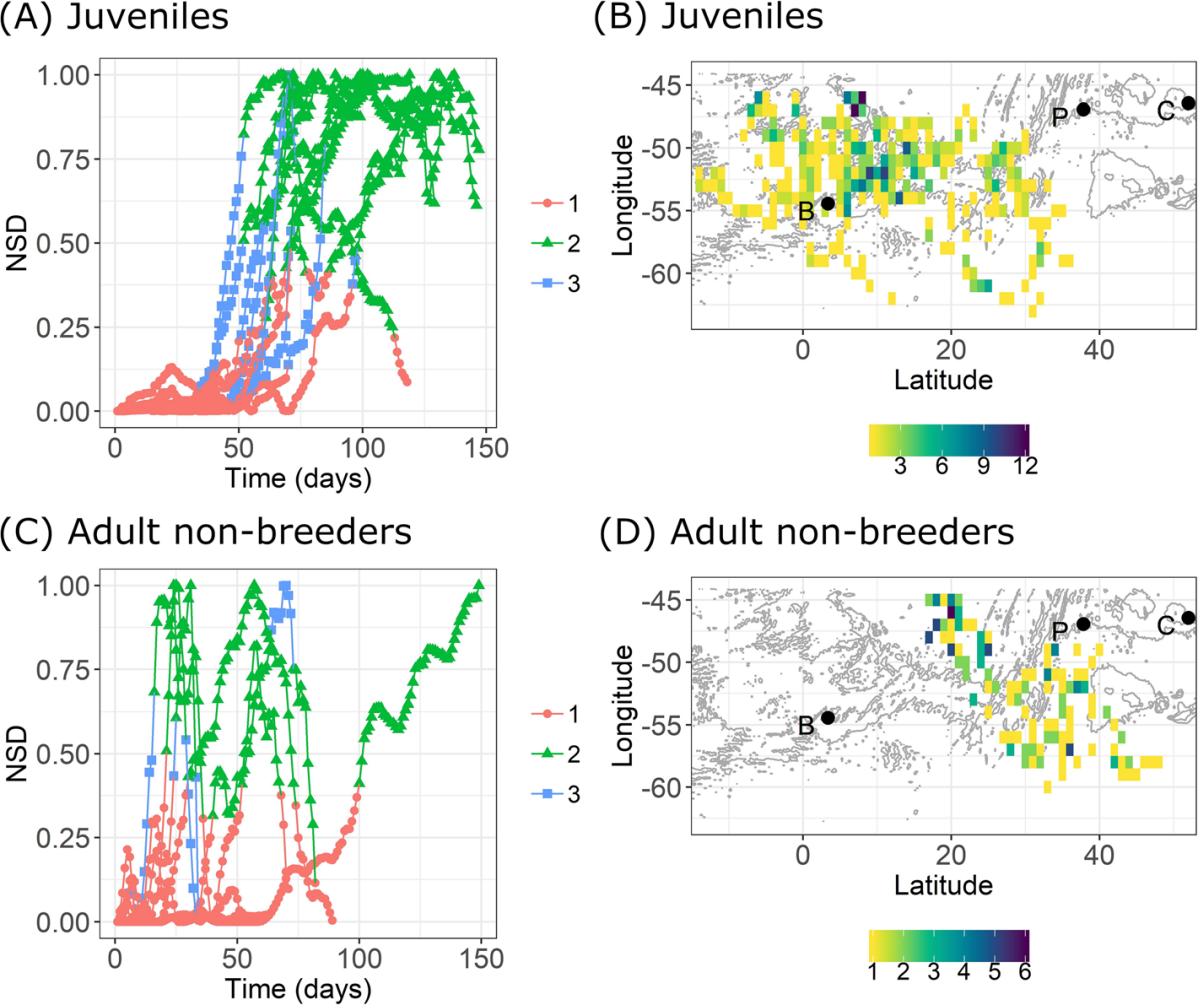
Net Squared Displacement (NSD) time series for juveniles (**a**, *N* = 12) and adult non-breeders (**c**, *N* = 6) according to three latent states (symbols 1–3). The right figures, **b** and **d**, show the corresponding daily locations of birds for ‘state 2’ (winter). The colour-coding indicates the number of days that birds spent within a grid of 1°× 1° (C: Crozet islands, P: Prince Edward islands, B: Bouvet island). Note that juveniles that had their tags stopped during the first 3–4 months at sea (*N* = 5, Additional file 1: Table S1) were not included in this analysis

When leaving their natal colony during late spring/early summer (November to January), juveniles predominately moved southward and generally remained within the vicinity of the Antarctic Polar Front (APF; Figs. 1 and 2). At the end of summer and the beginning of autumn (March), juveniles started to move south-westward with little directional variation between individuals (Fig. 2). This traveling direction was maintained throughout winter, during which juveniles ranged over a large geographical area (Figs. 1 and 3b). During late winter/early spring (between July and October), some juveniles (*N* = 5), changed direction and started their return phase towards their natal colony (Additional file 1: Figure S3). In comparison, although non-breeders also moved towards the south-west during autumn and remained within the same general area (APZ) as the juveniles, they ranged over a much smaller area and arrived much later in the season (June; Figs. 1 and 3d; Additional file 1: Figure S1). In addition, most non-breeders started their return phase towards the Crozet Archipelago during winter (between June and October, Additional file 1: Fig. S4).

NSD time series indicated 3 different phases for the movement patterns of both juveniles and non-breeders (Fig. 3a&c, see Additional file 1: Figure S5 for the five individuals that had notably shorter tracking durations): a first phase of encampment (‘state 1’), followed by a directed transiting phase (‘state 3’) which culminated in a final phase (‘state 2’) that could correspond to a wintering area. Both juveniles and non-breeders exhibited considerable inter-individual variation in the onset date of the final phase (‘state 2’, Fig. 3a&c) with daily locations of ‘state 2’ extending over a large geographic area (Fig. 3b&d). Moreover, PCA analysis showed that both juveniles and adults encountered a large range of environmental conditions, with considerable inter- and intra-individual variation exhibited by juveniles and inter-individual variation exhibited by non-breeders (Additional file 1: Figures S6 and S7). Hence, our analysis indicated that neither juveniles nor non-breeders reached a specific geographical wintering area.

### Travel versus foraging dives

Traveling speeds and dive parameters of birds were separated into three distinct clusters for both juveniles and non-breeding adults (Table 1, Additional file 1: Figure S8). The first cluster was interpreted to represent a ‘traveling’ dive mode as it was associated with shallow dive depths and fast traveling speeds. The second and the third clusters were associated with low traveling speeds and increased dive depths and durations and were, therefore, considered to represent a ‘foraging’ dive mode. Overall, the proportion of time spent in either traveling (30%) or foraging (70%) dive modes did not differ between juveniles and non-breeding adults (GLMM, Z = 0, *P* > 0.05, Table 1). Traveling and foraging dive modes occurred throughout the distributions of the birds (Additional file 1: Figure S9). However, the cluster analysis revealed that juveniles performed a higher proportion of traveling dives (70%) during their first week after leaving the colony, which was followed by an intense ~ three weeks period of foraging dives with little time spent traveling, after which time spent within the traveling dive mode increased progressively (Additional file 1: Figure S10). After ~ 18 weeks at sea, time spent traveling progressively decreased, in favour of foraging time. During winter, juveniles spent less time traveling than foraging (Additional file 1: Figure S10). Similarly, the time the non-breeders allocated to traveling was initially high and decreased over time, while the reverse was true for foraging (Additional file 1: Figure S10). Dive depth and duration during foraging dives was greater and surface interval between dives was shorter in non-breeders compared to that of juveniles (Table 1). In addition, traveling speed was greater in non-breeders than juveniles (Table 1).

**Table 1:** Summary of dive parameters and travel speed of juvenile and non-breeding adult king penguins

Stage	Cluster	Dive Depth (m)	Dive duration (s)	Surface duration (s)	Travel Speed (km/h)	Allocated time (Proportion)	Modes
Juveniles	1	37.1 ± 31.8	141.2 ± 56.6	41.4 ± 22.9	2.7 ± 0.7	0.3	traveling
	2	31.0 ± 28.9	120.7 ± 59.0	57.1 ± 40.1	1.1 ± 0.5	0.4	foraging
	3	144.7 ± 42.9	280.3 ± 59.2	97.9 ± 37.4	1.4 ± 0.8	0.3	foraging
Non-breeding Adults	1	37.2 ± 40.6	156.0 ± 65.9	38.6 ± 21.6	3.6 ± 0.9	0.3	traveling
	2	30.3 ± 34.6	123.5 ± 67.9	65.9 ± 49.0	1.4 ± 0.7	0.3	foraging
	3	170.8 ± 48.2	316.5 ± 58.0	90.3 ± 28.0	1.5 ± 0.9	0.4	foraging

Values are shown according to behavioral clusters; see Additional file 1: Figure S8 for visual illustration of the clusters, according to dive and travel parameters

### Habitat modeling (environmental features)

**Fig. 4:**
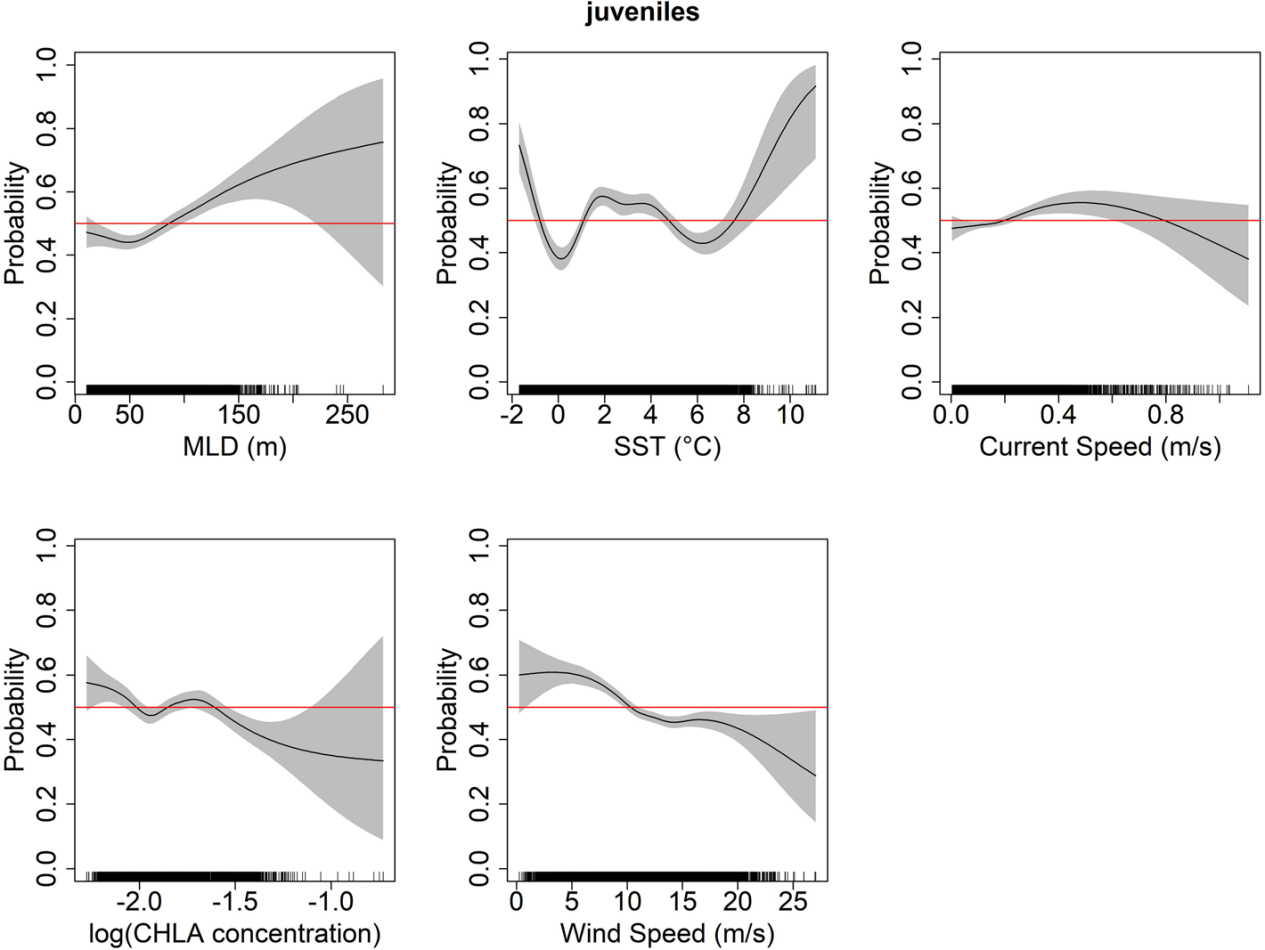
Relationship between the probability of switching dive modes (traveling mode, ‘0’ versus foraging mode, ‘1’) and various environmental variables (MLD: Mixed Layer Depth, SST: Sea Surface Temperature, Current speed, CHLA: Chlorophyll A concentration, and Wind speed) for juvenile king penguins (*N* = 17). Plots are based on GAMM estimates from the best model (see Additional file 1: Table S3 for best model outputs). The red line indicates the line of equality, when both behavioral modes are equally likely, illustrating the absence of an effect of the variable tested

**Fig. 5:**
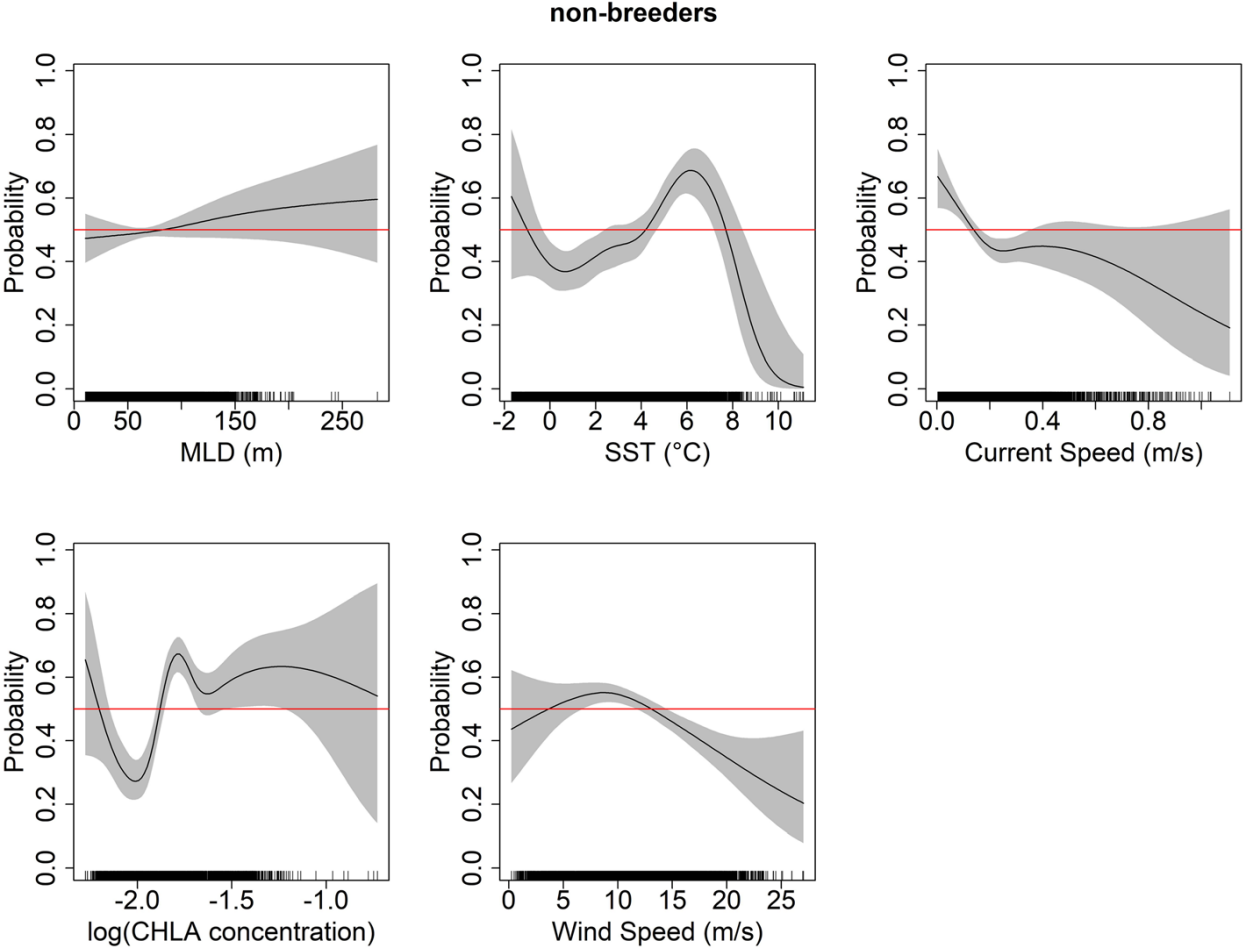
Relationship between the probability of switching dive modes (traveling mode, ‘0’ versus foraging mode, ‘1’) and various environmental variables (MLD: Mixed Layer Depth, SST: Sea Surface Temperature, Current speed, CHLA: Chlorophyll A concentration, and Wind speed) for **non-breeding** adult king penguins (N = 5). Plots are based on GAMM estimates from the best model (see Additional file 1: Table S3 for best model outputs). The red line indicates the line of equality, when both behavioral modes are equally likely, illustrating the absence of an effect of the variable tested

The effects of environmental variables on the probability to be in a foraging dive mode and not in a traveling dive mode (hereafter “foraging probability”) varied for both juveniles and non-breeders (GAMM analysis; Figs. 4 and 5). Overall, the foraging probability did not differ between juveniles and non-breeders and did also not differ between years (parametric coefficients in Additional file 1: Table S3). However, foraging probabilities differed significantly between seasons, that is, there was a greater probability for birds to be in a foraging dive mode during winter than during summer or autumn (see Additional file 1: Table S3).

For juveniles foraging probabilities increased when the mixed layer depth (MLD) exceeded 150 m (Fig. 4) but this was not significant for non-breeders (Additional file 1: Table S3). For juveniles, foraging probability increased at three distinct sea surface temperature ranges (SST): when SST was < 1 °C, which corresponds to the limit of the sea ice zone; between 2 °C and 5 °C, which corresponds to the APZ, and, lastly, when SST was > 8 °C, which corresponds to the SAF. In comparison, the foraging probability of non-breeders increased at SST between 4 °C and 6 °C (corresponding to the APF) and also when SST was < 0 °C. Both juveniles and non-breeding adults were more likely to forage at lower current and wind speeds. Finally, foraging probability of non-breeders increased with increasing CHLA concentrations, while juveniles were more likely to travel at comparable CHLA concentrations (Figs. 4 and 5).

**Fig. 6:**
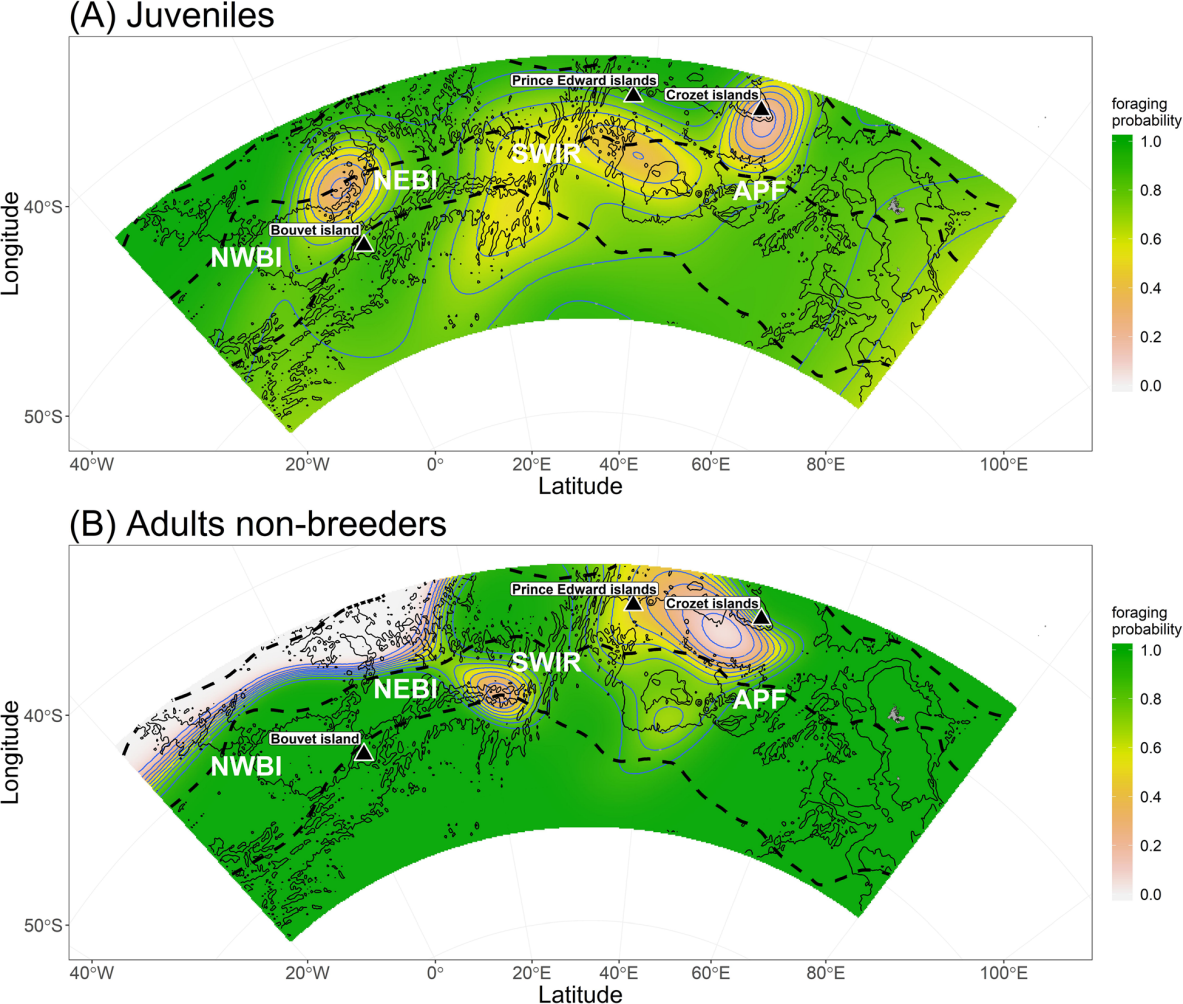
Spatial prediction maps for juveniles (**a**) and non-breeding adults (**b**), indicating the probability of foraging (best GAMM model). Green indicates a high probability of foraging (i.e. a low probability of traveling), while white indicates a low probability of foraging. The blue iso-lines correspond to the probabilities of foraging (ranging from 0 to 1). The dashed lines indicate the average position of major oceanic fronts within the area (averaged over the two monitoring years); from top to bottom: Sub-Antarctic Front, Antarctic Polar Front, and South of Antarctic Circumpolar Current Front. Capital letters indicate key oceanic areas; from left to right: northwest of Bouvet Island (NWBI), northeast of Bouvet Island (NEBI), Southwest Indian Ridge (SWIR), and Antarctic Polar Front (APF). Background lines indicate the bathymetry, while sub-Antarctic islands are marked by black dots

Prediction maps of the foraging probability (Fig. 6) illustrated that in some areas both juveniles and non-breeders were more likely to travel, which was especially the case around the Crozet and Prince Edward archipelagos and along the Southwest Indian Ridge (SWIR). By contrast, areas to the south and especially to the northeast and northwest of Bouvet Island were more likely associated with foraging in juveniles, while non-breeders were more likely to forage south of the APF and north of the SWIR (Fig. 6).

### Orientation preferences in relation to oceanic current and wind

**Fig. 7:**
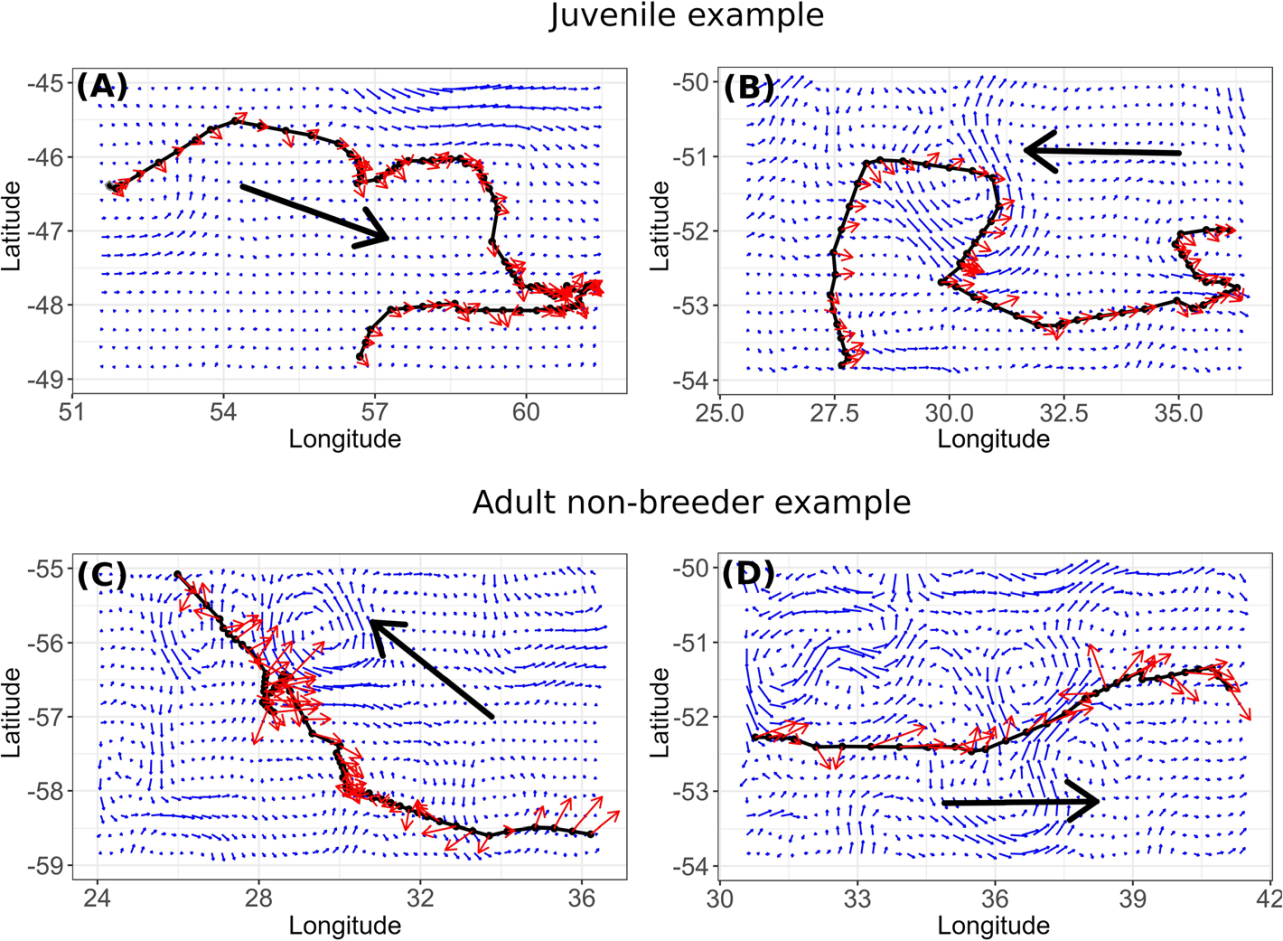
Reconstructed tracks (black lines, with the black arrow indicating the direction of movement) for a juvenile (Top, **a** & **b**) and non-breeding adult king penguin (Bottom, **c** & **d**) projected on top of maps indicating current speed and direction (blue arrows; speed indicated by arrow length), which were averaged over the relevant periods. Red arrows indicate wind speed (longer arrows correspond to a greater wind speed) and direction, extracted for each location during the relevant period. (**a**) corresponds to the departure of a juvenile penguin from Possession Island (Crozet Archipelago; black dot on the left) when the bird traveled through a relatively low current speed area and initially moved with the wind direction, before turning against it. (**b**) Later on, the same individual moved against the current and the wind for the majority of the period shown. (**c**) For comparison, here a non-breeding adult moved against the current and wind for most part of the period shown but traveled in the same direction as current and wind during its return phase to the natal colony (**d**)

While the ACC generally flows eastwards, all birds mainly moved towards the south-west/west, particularly during autumn (Fig. 1). Movement occurred both against and with prevailing wind and current directions (Fig. 7). However, birds most frequently adopted headings resulting in perpendicular orientation with respect to direction of the wind and current (~ 50% for each season and for both juveniles and non-breeders; Table 2). Furthermore, orientation of birds relative to current and wind directions differed between seasons (Table 2). During autumn, juvenile orientation was more frequently against the wind and current directions, while the reverse was true during winter (Table 2). For non-breeders, the pattern was less obvious during autumn, however during winter, orientation was more frequently with the wind and current directions than against it, as observed for juveniles (Table 2).

**Table 2:** Proportion (% ± SD) of tracks, when birds moved against, with, or perpendicular to the direction of the wind/ocean current across seasons

Medium	Stage	Direction	Summer	Autumn	Winter
Wind	juveniles	Against	27 ± 4^c^	32 ± 3^d^	20 ± 4^b^
		Similar	23 ± 3^bc^	15 ± 3^a^	28 ± 4^cd^
		Cross	49 ± 4^e^	53 ± 4^ef^	52 ± 4^ef^
	non-breeding adults	Against	–	23 ± 3^bc^	18 ± 3^ab^
		Similar	–	19 ± 2^ab^	54 ± 4^ef^
		Cross	–	58 ± 3^f^	28 ± 3^cd^
Ocean Current	juveniles	Against	28 ± 4^cd^	29 ± 3^d^	17 ± 3^a^
		Similar	23 ± 3^b^	20 ± 3^ab^	35 ± 4^e^
		Cross	49 ± 4^f^	51 ± 4^f^	47 ± 4^f^
	non-breeding adults	Against	–	29 ± 3^cde^	18 ± 3^ab^
		Similar	–	23 ± 3^abc^	29 ± 3^cde^
		Cross	–	48 ± 3^f^	52 ± 4^f^

As non-breeding adults left long after the juveniles, sufficient summer data for them is lacking. Differences between stage (juveniles/non-breeding adults), direction and season were assessed with 2 different GLMMs per medium (wind or current) with ‘proportion’ as response variable, using a binomial family. Cells that share the same letter per type of medium (wind or current) are not significantly different from each other. The Bonferroni procedure was used for multiple comparisons

**Fig. 8:**
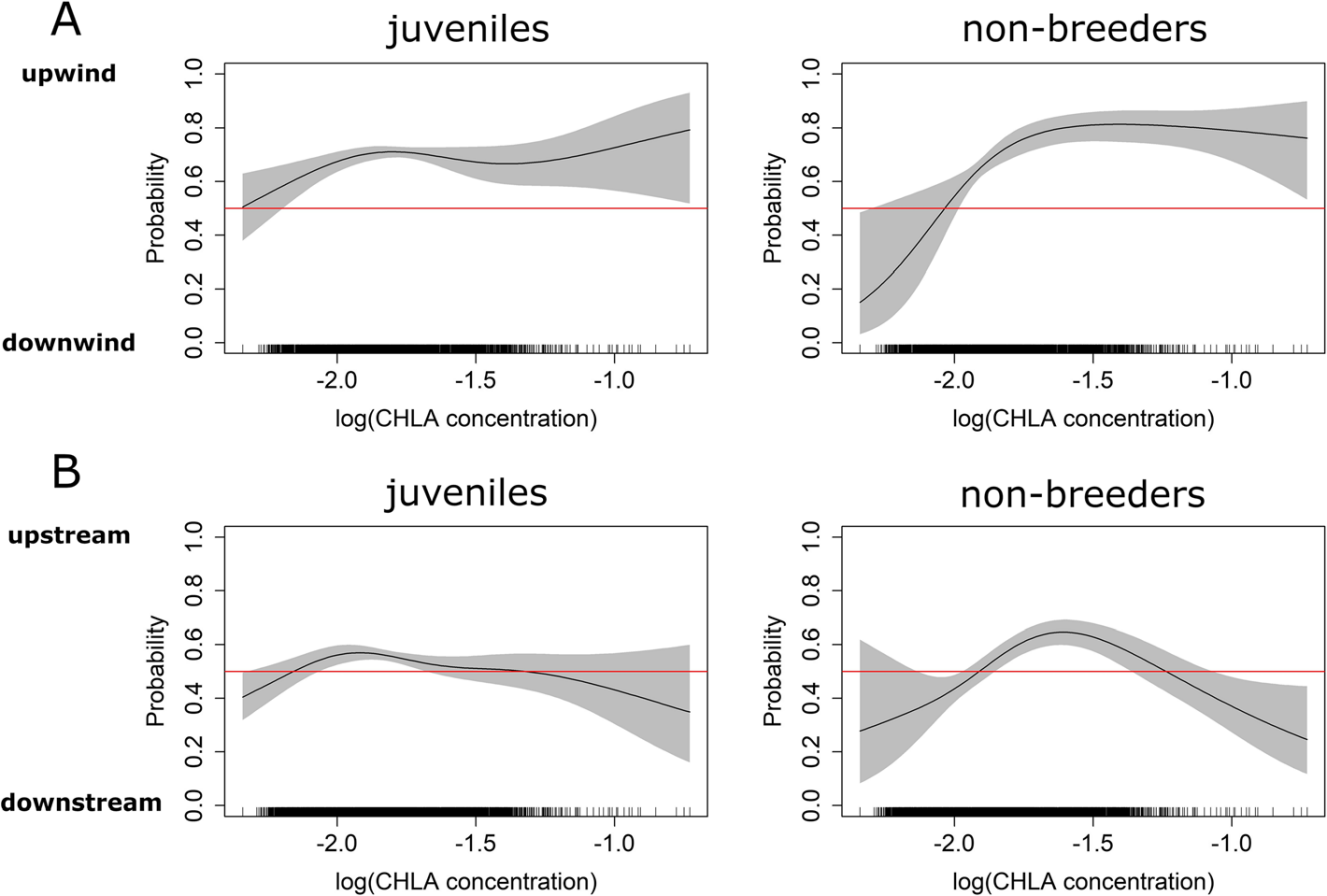
Relationship between the probability of moving against or with the wind (**a**) and against or with the current (**b**) in relation to the CHLA concentration of the next location (t + 1) along the movement trajectories of juvenile (left) and non-breeding adult king penguins (right). Plots are based on GAMM estimates (see Additional file 1: Table S4 and S5 for model outputs). The red line indicates the line of equality, when both behavioral modes are equally likely, illustrating the absence of an effect of the variable tested

Regarding the effect of ocean productivity on bird orientation, models showed that both juveniles and non-breeders were more likely to move against the wind, when CHLA concentration at their next location was higher than their previous one (Fig. 8a). However, no clear pattern emerged concerning the effect of CHLA concentration on bird orientation with respect to prevailing currents (Fig. 8b).

## Discussion

In our study we (1) investigated the movement patterns and foraging behavior of juvenile king penguins during their first year at sea, and (2) simultaneously tracked non-breeding adults to allow for a direct comparison. The extended deployment duration of tags (transmission of location and dive parameters for up to ~ 300 days, see Additional file 1: Table S1) provided the opportunity to study both the ontogeny of juvenile movements and associated foraging behavior in great detail.

### First juvenile movements: a seasonal migration?

Net Square Displacement (NSD) time series analysis showed that juvenile movement patterns could be divided into three distinct phases: (1) a first initial dispersion, with juveniles predominately moving south-west and south-east of the Crozet islands within the vicinity of APF; (2) a departure from these summer feeding grounds continuing in a south-west direction; (3) a exploration phase during winter, when juveniles range over a large area (Fig. 3b), with variable environmental conditions (Additional file 1: Figure S6). Overall, the movement patterns of juvenile king penguins for their first year at sea cannot be considered as a seasonal migration in a strict sense, since they did not reach a particular wintering area. Instead, the definition may fall somewhere between the two following extremes: (1) a strictly exploratory or nomadic behavior and (2) a truly migratory behavior, characterized by straight movements along a corridor, with animals reaching well defined wintering areas [54].

#### Summer

Upon leaving their natal colony in austral summer, juvenile birds prospected over a large area but generally remained within the vicinity of the APF (Figs. 1 and 2), where resource availability is high during summer [55]. During this period, this area is the main foraging zone for a large number of marine predators [55], including breeding king penguins [15]*.* Thus, juveniles may have followed breeding adults which departed from the same colony. The existence of such a pattern has been shown in other bird species, like boobies [56] and storks [57].

#### Autumn

After the initial general southward movement from the Crozet Archipelago, juvenile penguins moved south-westward in March, at the end of the summer and the beginning of autumn (Figs. 1 and 2). This movement was likely motivated by a decline in food resources within the PFZ. Such resource decline might be caused by a vertical migration of prey to greater depth, reducing prey availability to penguins [58, 59] and/or by inter-specific competition [55]. The latter might explain why we found reduced foraging probabilities for birds in the vicinity of the Crozet and Prince Edward islands (Fig. 6), which both host large marine predator populations. The movement of juvenile king penguins during this time may have also been motivated by their desire to occupy foraging habitat with less competitors, leading, in turn, to a niche segregation between juveniles and breeding adults from Crozet [15] and Prince Edward [60] islands, which has been observed in many species [61]. Interestingly, adult non-breeders also moved south-west in autumn and foraged in the same general area as juveniles (APZ, Fig. 1, Additional file 1: Figure S1). However, they arrived in the area after the juveniles. Hence, juvenile king penguins managed to find these areas without following adult birds from the Crozet islands (breeders do not frequent these areas and non-breeders arrived after the juveniles). However, breeding king penguins from Marion Island (Prince Edward Archipelago) are known to visit this zone [60] and the juveniles may have encountered these conspecifics. Surprisingly, both juveniles and non-breeding adults tended to either avoid the Southwest Indian Ridge (SWIR) altogether or traversed it (increased traveling probability; Fig. 6). The SWIR is known for its high productivity due to the interaction between its prominent bathymetric features and currents in the area [62]. Its importance as a foraging habitat to other marine predators has been well documented e.g. [63]. However, a recent study that tracked breeding king penguins from Marion Island during winter also found that birds rapidly traveled through the area of the SWIR and generally avoided areas with a high eddy kinetic energy, such as found near the SWIR [60].

#### Winter

During winter, the juvenile king penguins continued their south-westward movement and ranged over a large geographic area (Figs. 1 and 3b). When juveniles reached their large foraging areas in winter (Fig. 3b), the probabilities to be in a foraging dive mode (versus traveling dive mode) was high in specific places (Fig. 6). These areas were situated to the general south and especially to the northeast/northwest of Bouvet Island (Fig. 6) and are known to be of great importance for foraging marine predators, such as penguins and fur seals [64]. In contrast, while non-breeding adults also foraged mainly within the PFZ and AZ during winter (Figs. 1, 3d and 6), most birds did not move as far west as the SWIR. However, two non-breeding adults went as far north as the SAF (Fig. 1, Additional file 1: Figure S1), where estimated foraging probability was high (Fig. 6). Previously, both breeders and failed-breeders from the Crozet islands have also been reported to forage within the AZ during winter [65, 66], and within the SAF [66].

### Reaching foraging areas and at-sea survival during the first year

During their early life at sea, juvenile king penguins face a multitude of challenges related to the development of sufficient behavioral and physiological capacities [4]. It has been suggested that this initial period of their oceanic existence is energetically challenging [26] and might, consequently, lead to increased mortality, especially during the beginning of autumn when prey becomes less abundant or accessible [21]. In our study, five juvenile penguins had exceptionally short monitoring durations and distances traveled compared to any other penguin within the study (Additional file 1: Table S1). These individuals likely died at sea probably because of insufficiently developed diving and foraging skills [21], which lead to inadequate food intake and a negative energy balance, exacerbated by an insufficient body insulation (elevated thermoregulatory costs), to which birds eventually succumbed [21, 26]. This suggests that reaching profitable winter foraging areas (around Bouvet Island), is crucial for juvenile king penguin survival and might explain the relatively high survival rate during their first year at sea (68–87%) [30]. For comparison, the survival rate of juvenile emperor penguins *Aptenodytes forsteri* is considerably lower, at ~ 40% [67].

### Orientation: innate behavior, environmental cues and adult conspecifics

The observed movement trajectories of juvenile king penguins raise the question of how birds were able to orient themselves and find profitable foraging areas. We suggest that there are three possible, non-exclusive explanations: 1) juveniles rely on environmental cues to find profitable foraging areas, 2) they follow adult conspecifics, especially during their initial dispersion towards the APF and/or 3) their general movement pattern, at least in part, is inherited.

During the last glaciation period, which ended ~ 18,000 years ago, presumably few refuges existed for king penguins (e.g. Gough Island, the Falkland Islands and New Zealand), as they require ice-free land to breed [68]. At the end of this last glaciation period, when the climate warmed, the global population of king penguins increased considerably, as the retreating sea ice opened up new breeding sites and migration corridors towards new feeding areas [68]. Accordingly, given such a scenario, it is possible that juvenile penguins depend to some degree on innate direction preferences during their dispersion. It might be possible that juveniles found their profitable foraging areas during autumn and winter by using a spatio-temporal program (“clock and compass” concept [69]). Migration or dispersal directions have been shown to be partially pre-determined in a number of animals, for example in juvenile flying seabirds [14, 70], sea turtles [7], seals [71]. In our study, movement trajectories of juvenile king penguins were directed and synchronized (Fig. 2), similar to what has been reported for juvenile king penguins from South Georgia and the Falkland Islands [19] and for juvenile elephant seals [72]. The very directed movements southward and then south-westward of juvenile king penguins during summer and autumn/winter, respectively, and the large distances covered, support the hypothesis that their first movements are based, at least partially, on innate behavior.

However, it is likely that birds require additional cues, such as provided by the environment (e.g. CHLA, reflecting ocean productivity) or adult conspecifics, to find profitable foraging areas, especially on a smaller scale. In our track analysis we investigated to what degree the trajectories of both juvenile and non-breeding adult king penguins correlated with the prevailing current and wind directions (Table 2). During autumn, when both groups traveled towards the south-west, orientation was predominately perpendicular and against the wind and current directions, while during winter, when birds started their return to the Crozet islands, they mostly traveled perpendicular and with the wind and current directions (Fig. 7). Moreover, our analysis showed that both bird groups where more likely to travel when encountering strong winds or currents (Figs. 4 and 5), when cues from these media may be amplified. In addition, orientation of both groups was increasingly directed upwind when CHLA concentrations increased along the track (Fig. 8).

When traveling, birds have to surface frequently to breathe, hence, they may be able to pick up chemical cues from the air, e.g. Dimethyl sulphide (DMS). However, the capacity for chemical detection by penguins is largely unexplored, debated [73], and at-sea studies are needed to confirm that they can use DMS as a cue at the surface [74]. Nevertheless, recent studies suggest that king penguins can detect naturally occurring sulphur compounds that are associated with primary production [75]. Hence, it seems likely that wind could relay chemical information from areas of high productivity to penguins [74, 76, 77] and this could be a mechanism for birds to find favourable foraging areas. The use of wind-borne cues has also been suggested for other pelagic animals, like marine turtles [78], which showed no reliance on current-borne cues for orientation [79–81] (but see [82]).

Like all pelagic animals, penguins moving within deep oceans lack stationary reference points during movement, which are typically provided by the ocean floor. They are therefore unable to sense current velocity [83]. Thus, the orientation of king penguins with respect to current, which we observed, might have been coincidental. Moreover, it is not known whether juvenile king penguins, during their first movement away from the colony in summer, are accompanied by adult conspecifics.

In conclusion, the directed movement of juveniles during autumn, when birds headed towards the south-west, suggests that such movement might be pre-determined (i.e. inherited), at least partially. At a smaller scale, birds may follow environmental gradients [84]. To what degree adults and juvenile penguins might also be able to make use of a geo-magnetic or celestial compass for navigation and orientation remains to be investigated [83].

### The relevance of juvenile exploratory behavior

The exploratory behavior of juvenile king penguins which we observed during their first year at sea might enable juveniles to learn the spatial location of important foraging areas, far away from future breeding sites, and might be critical for their survival. Such memory based foraging strategies [10] might explain the extended periods of immaturity and the large distribution ranges of juveniles that have also been observed in other seabird species e.g. [12]. Hence, this exploratory behavior might be important for juvenile survival and, ultimately, population survival.

Non-breeding adults used the same travel direction as juveniles and reached the same general foraging areas. Hence, they might have also once learned navigation to these areas during the first year of their life at sea [85]. However, since these adults likely plan to breed during the next season (November of the same year that they were instrumented), their roaming behavior was certainly more constrained than that of juveniles, albeit much less compared to adult breeders. Accordingly, these non-breeding adults did not move as far to the west or south as juveniles. In this context, it would be very interesting to monitor juveniles, during their second or third year at-sea, to determine the importance of a learning process for the establishment of their movement and migration patterns [7, 10].

The exploratory behavior we observed in the juvenile king penguins from the Crozet Archipelago might be typical for juveniles of this species. The genetic differentiation among king penguin colonies in the sub-Antarctic is small, despite the great distances between them, suggesting that natal dispersal between islands is frequent [86]. However, this is in contradiction with the observed strong philopatry of adult breeders in this species [87]. Hence, juveniles are the likely candidates to facilitate gene flow amongst different king penguin colonies [86].

## Conclusions and outlook

Our study investigated both the movement patterns and the foraging behavior of juvenile penguins during their first year at sea, and simultaneously tracked non-breeding adults for a direct comparison. The satellite relay tags allowed us to study both the ontogeny of juvenile dispersion and associated foraging behavior in great detail. In summary, our study suggests that: (1) the dispersion of juvenile king penguins falls between the extremes of (a) strictly explorative/nomadic behavior and (b) truly migratory behaviour; (2) both innate and learning processes could be involved in their movements; (3) penguins may use information conveyed by winds (rather than currents) to detect productive foraging areas but their orientation with respect to the media (wind/current) might also be coincidental.

The distribution and survival of juvenile king penguins is of key importance for the future development of their populations. To anticipate the consequences of rapid climate change on king penguin populations, a clear understanding of juvenile dispersion, survival, and their use of winter foraging areas is critical. The position of the APF, a key foraging area for king penguins during the summer is predicted to shift increasingly towards the south during coming decades [24], thus increasing distance between their colonies and foraging areas, which will have negative effects on these birds’ breeding success [15]. Furthermore, understanding how juvenile wintering foraging habitats might change over the next century will be crucial. Further studies should evaluate to what degree penguins will be able to adjust their foraging movements when confronted with rapid change. A pre-determined, rigid movement pattern of juveniles might lead populations into an ecological trap, with a strong negative impact on recruitment [20, 23].

## Supplementary information

**Additional file 1.** Supplementary Tables and Figures  about  individuals  tracking parameters,  models outputs,  distribution  maps,  PCA of environmental variables during the  winter,  clustering  outputs  and  time series.

## Data Availability

All dive and location data analyzed during the current study are available from the corresponding author on request.

## Abbreviations

ACC: Antarctic Circumpolar Current
APF: Antarctic Polar Front
AZ: Antarctic Zone
CHLA: Chlorophyll A concentration
GAMM: Generalized Additive Mixed-effects Model
GLMM: Generalized Additive Mixed Model
LME: Linear Mixed Effect
MLD: Mixed Layer Depth
NEBI: Northeast of Bouvet Island
NWBI: Northwest of Bouvet Island
PCA: Principal Component Analysis
PFZ: Polar Frontal Zone
SACCF: Southern Antarctic Circumpolar Current Front
SACZ: Southern Antarctic Circumpolar Zone
SAF: Sub-Antarctic Front
SIC: Sea Ice Concentration
SSH: Sea Surface Height
SST: Sea Surface Temperature
SWIR: Southwest Indian Ridge

## References

[1] Lack DL (1954). The natural regulation of animal numbers.

[2] Levitis DA (2011). Before senescence: the evolutionary demography of ontogenesis. Proc R Soc Lond B Biol Sci.

[3] Wunderle JM. Age-specific foraging proficiency in birds. Curr Ornithol. Plenum Press. New York, NY: Plenum Press; 1991. p. 273–324.

[4] Marchetti K, Price T (1989). Differences in the foraging of juvenile and adult birds: the importance of developmental constraints. Biol Rev.

[5] Dukas R, Ratcliffe JM (2009). Cognitive Ecology II.

[6] Martin K (1995). Patterns and mechanisms for age-dependent reproduction and survival in birds. Am Zool.

[7] Scott R, Marsh R, Hays GC (2014). Ontogeny of long distance migration. Ecology..

[8] Phillips R, Lewis S, González-Solís J, Daunt F. Constraint, compromise and carry-over: causes and consequences of individual variability and specialization in foraging and migration patterns of seabirds. Mar Ecol Prog Ser. 2017;578.

[9] Hays GC, Ferreira LC, Sequeira AMM, Meekan MG, Duarte CM, Bailey H (2016). Key questions in marine megafauna movement ecology. Trends Ecol Evol.

[10] Fagan WF, Lewis MA, Auger-Méthé M, Avgar T, Benhamou S, Breed G (2013). Spatial memory and animal movement. Clobert J, editor. Ecol Lett.

[11] Hazen EL, Maxwell SM, Bailey H, Bograd SJ, Hamann M, Gaspar P (2012). Ontogeny in marine tagging and tracking science: technologies and data gaps. Mar Ecol Prog Ser.

[12] Grecian WJ, Lane JV, Michelot T, Wade HM, Hamer KC (2018). Understanding the ontogeny of foraging behaviour: insights from combining marine predator bio-logging with satellite-derived oceanography in hidden Markov models. J R Soc Interface.

[13] Debeffe L, Morellet N, Cargnelutti B, Lourtet B, Coulon A, Gaillard JM (2013). Exploration as a key component of natal dispersal: dispersers explore more than philopatric individuals in roe deer. Anim Behav.

[14] de Grissac S, Börger L, Guitteaud A (2016). Weimerskirch H.

[15] Bost CA, Cotté C, Terray P, Barbraud C, Bon C, Delord K (2015). Large-scale climatic anomalies affect marine predator foraging behaviour and demography. Nat Commun.

[16] Wilson RP (2004). Antennae on transmitters on penguins: balancing energy budgets on the high wire. J Exp Biol.

[17] Bannasch R, Wilson RP, Culik B (1994). Hydrodynamic aspects of design and attachment of a back-mounted device in penguins. J Exp Biol.

[18] Thiebot J-B, Lescroël A, Barbraud C, Bost C-A (2013). Three-dimensional use of marine habitats by juvenile emperor penguins Aptenodytes forsteri during post-natal dispersal. Antarct Sci.

[19] Pütz K, Trathan PN, Pedrana J, Collins MA, Poncet S, Lüthi B (2014). Post-fledging dispersal of king penguins (*Aptenodytes patagonicus*) from two breeding sites in the South Atlantic. PLoS One.

[20] Sherley RB, Ludynia K, Dyer BM, Lamont T, Makhado AB, Roux J-P (2017). Metapopulation tracking juvenile penguins reveals an ecosystem-wide ecological trap. Curr Biol.

[21] Orgeret F, Weimerskirch H, Bost C-A (2016). Early diving behaviour in juvenile penguins: improvement or selection processes. Biol Lett.

[22] de Brooke M (2004). L. the food consumption of the world’s seabirds. Proc R Soc Lond B Biol Sci.

[23] Le Bohec C, Durant JM, Gauthier-Clerc M, Stenseth NC, Park Y-H, Pradel R (2008). King penguin population threatened by Southern Ocean warming. Proc Natl Acad Sci.

[24] Cristofari R, Liu X, Bonadonna F, Cherel Y, Pistorius P, Maho YL (2018). Climate-driven range shifts of the king penguin in a fragmented ecosystem. Nat Clim Chang.

[25] Ponganis PJ, Starke LN, Horning M, Kooyman GL (1999). Development of diving capacity in emperor penguins. J Exp Biol.

[26] Enstipp MR, Bost C-A, Bohec CL, Bost C, Maho YL, Weimerskirch H (2017). Apparent changes in body insulation of juvenile king penguins suggest an energetic challenge during their early life at sea. J Exp Biol.

[27] Labrousse S, Orgeret F, Solow AR, Barbraud C, Bost CA, Sallée J-B (2019). First odyssey beneath the sea ice of juvenile emperor penguins in East Antarctica. Mar Ecol Prog Ser.

[28] Bost C-A, Delord K, Barbraud C, Cherel Y, Pütz K, Cotté C (2013). King Penguin. Penguins: natural history and conservation.

[29] Delord K, Barbraud C, Weimerskirch H (2004). Long-term trends in the population size of king penguins at Crozet archipelago: environmental variability and density dependence?. Polar Biol.

[30] Saraux C, Viblanc VA, Hanuise N, Le Maho Y, Le Bohec C (2011). Effects of individual pre-fledging traits and environmental conditions on return patterns in juvenile king penguins. PLoS One.

[31] Le Bohec C, Gauthier-Clerc M, Grémillet D, Pradel R, Béchet A, Gendner J-P (2007). Population dynamics in a long-lived seabird: I. impact of breeding activity on survival and breeding probability in unbanded king penguins. J Anim Ecol.

[32] Madec G. the Nemo team (2008) NEMO Ocean engine. Note Pôle Modélisation Institut Pierre-Simon Laplace IPSL Fr 2008;

[33] Rohr Tyler, Long Matthew C., Kavanaugh Maria T., Lindsay Keith, Doney Scott C. (2017). Variability in the mechanisms controlling Southern Ocean phytoplankton bloom phenology in an ocean model and satellite observations. Global Biogeochemical Cycles.

[34] Venables H, Meredith MP, Atkinson A, Ward P (2012). Fronts and habitat zones in the Scotia Sea. Deep Sea Res Part II Top Stud Oceanogr.

[35] Wickham H, Chang W, R Studio. ggplot2: Create elegant data visualisations using the grammar of graphics. 2016. https://cran.r-project.org/web/packages/ggplot2/index.html

[36] Freitas C, Lydersen C, Fedak MA, Kovacs KM (2008). A simple new algorithm to filter marine mammal Argos locations. Mar Mammal Sci.

[37] Cotté C, Park Y-H, Guinet C, Bost C-A (2007). Movements of foraging king penguins through marine mesoscale eddies. Proc R Soc B Biol Sci.

[38] Gaspar P, Georges J-Y, Fossette S, Lenoble A, Ferraroli S, Maho YL (2006). Marine animal behaviour: neglecting ocean currents can lead us up the wrong track. Proc R Soc Lond B Biol Sci.

[39] Bastille-Rousseau G, Potts JR, Yackulic CB, Frair JL, Ellington EH, Blake S (2016). Flexible characterization of animal movement pattern using net squared displacement and a latent state model. Mov Ecol.

[40] Jorgensen SJ, Arnoldi NS, Estess EE, Chapple TK, Rückert M, Anderson SD (2012). Eating or meeting? Cluster analysis reveals intricacies of white shark (*Carcharodon carcharias*) migration and offshore behavior. PLoS One.

[41] Husson F, Josse J, Le S (2017). Mazet J.

[42] VanDerWal J, Falconi L, Januchowski S. Storlie LS and C. SDMTools: Species Distribution Modelling Tools: tools for processing data associated with species distribution modelling exercises. 2014; https://cran.r-project.org/web/packages/SDMTools/index.html.

[43] Wood S. mgcv: Mixed GAM Computation vehicle with automatic smoothness estimation. 2017. https://cran.r-project.org/web/packages/mgcv/index.html

[44] Kato A, Ropert-Coudert Y, Ryan PG, Whitehead TO (2016). Habitat use and diving behaviour of macaroni Eudyptes chrysolophus and eastern rockhopper *E chrysocomefilholi* penguins during the critical pre-moult period. Mar Biol.

[45] Dormann CF, McPherson JM, Araújo MB, Bivand R, Bolliger J, Carl G (2007). Methods to account for spatial autocorrelation in the analysis of species distributional data: a review. Ecography..

[46] Wood S. Generalized additive models: an introduction with R: CRC press; 2006.

[47] Barton K (2009). MuMIn: multi-model inference.

[48] Burnham KP, Anderson DR (2002). Model selection and multimodel inference: a practical information-theoretic approach.

[49] Lund U, Agostinelli C, Arai H, Gagliardi A, Portugues EG, Giunchi D (2017). circular: Circular Statistics.

[50] Zeileis A, Grothendieck G, Ryan JA, Ulrich JM, Andrews F. zoo: S3 Infrastructure for regular and irregular time series (Z’s ordered observations). 2017. https://cran.r-project.org/web/packages/zoo/index.html

[51] Bates D, Maechler M, Bolker B, Walker S, Christensen RHB, Singmann H (2017). lme4: Linear Mixed-Effects Models using “Eigen” and S4.

[52] Lenth R, Love J. lsmeans: Least-Squares Means. 2017. from: https://cran.r-project.org/web/packages/lsmeans/index.html

[53] Bretz F, Hothorn T (2010). Westfall P.

[54] Jonzén N, Knudsen E, Holt RD, Sæther B (2011). Uncertainty and predictability: the niches of migrants and nomads.

[55] Bost CA, Cotté C, Bailleul F, Cherel Y, Charrassin JB, Guinet C (2009). The importance of oceanographic fronts to marine birds and mammals of the southern oceans. J Mar Syst.

[56] Yoda K, Murakoshi M, Tsutsui K, Kohno H (2011). Social interactions of juvenile brown boobies at sea as observed with animal-borne video cameras. PLoS One.

[57] Rotics S, Kaatz M, Resheff YS, Turjeman SF, Zurell D, Sapir N (2016). The challenges of the first migration: movement and behaviour of juvenile vs. adult white storks with insights regarding juvenile mortality. J Anim Ecol.

[58] Koslov AN, Shust KV, Zemsky AV. Seasonal and interannual variability in the distribution of *Electrona carlsbergi* in the southern polar front area. Sel Sci Pap Comm Conserv Antarct Living Resour CCAMLR Hobart. 1991:320–37.

[59] Charrassin J-B, Maho YL, Bost C-A (2002). Seasonal changes in the diving parameters of king penguins (*Aptenodytes patagonicus*). Mar Biol.

[60] Pistorius P, Hindell M, Crawford R, Makhado A, Dyer B, Reisinger R (2017). At-sea distribution and habitat use in king penguins at sub-Antarctic Marion Island. Ecol Evol.

[61] Matthysen E. Multicausality of dispersal: a review. Dispersal Ecol Evol. 2012:3–18.

[62] Ansorge IJ, Lutjeharms JRE (2003). Eddies originating at the south-west Indian ridge. J Mar Syst.

[63] Nel D, Lutjeharms J, Pakhomov E, Ansorge I, Ryan P, Klages N (2001). Exploitation of mesoscale oceanographic features by grey-headed albatross *Thalassarche chrysostoma* in the southern Indian Ocean. Mar Ecol Prog Ser.

[64] Blanchet M-A, Biuw M, Hofmeyr GG, de Bruyn PN, Lydersen C, Kovacs KM (2013). At-sea behaviour of three krill predators breeding at Bouvetøya—Antarctic fur seals, macaroni penguins and chinstrap penguins. Mar Ecol Prog Ser.

[65] Bost CA, Charrassin JB, Clerquin Y, RopertCoudert Y, Maho YL (2004). Exploitation of distant marginal ice zones by king penguins during winter. Mar Ecol Prog Ser.

[66] Cherel Y, Parenteau C, Bustamante P, Bost C-A (2018). Stable isotopes document the winter foraging ecology of king penguins and highlight connectivity between subantarctic and Antarctic ecosystems. Ecol Evol..

[67] Abadi F, Barbraud C, Gimenez O (2017). Integrated population modeling reveals the impact of climate on the survival of juvenile emperor penguins. Glob Change Biol.

[68] Trucchi E, Gratton P, Whittington JD, Cristofari R, Maho YL, Stenseth NC (2014). King penguin demography since the last glaciation inferred from genome-wide data. Proc R Soc B.

[69] Berthold P (1991). Orientation in birds.

[70] Yoda K, Yamamoto T, Suzuki H, Matsumoto S, Müller M, Yamamoto M (2017). Compass orientation drives naïve pelagic seabirds to cross mountain ranges. Curr Biol.

[71] Bornemann H, Kreyscher M, Ramdohr S, Martin T, Carlini A, Sellmann L (2000). Southern elephant seal movements and Antarctic Sea ice. Antarct Sci.

[72] Tosh CA, de Bruyn PJN, Steyn J, Bornemann H, van den Hoff J, Stewart BS (2015). The importance of seasonal sea surface height anomalies for foraging juvenile southern elephant seals. Mar Biol.

[73] Lu Q, Wang K, Lei F, Yu D, Zhao H (2016). Penguins reduced olfactory receptor genes common to other waterbirds. Sci Rep.

[74] Wright KLB, Pichegru L, Ryan PG (2011). Penguins are attracted to dimethyl sulphide at sea. J Exp Biol.

[75] Cunningham GB, Leclaire S, Toscani C, Bonadonna F (2017). Responses of king penguin Aptenodytes patagonicus adults and chicks to two food-related odours. J Avian Biol.

[76] Culik B, Hennicke J, Martin T (2000). Humboldt penguins outmanoeuvring El Nino. J Exp Biol.

[77] Amo L, Rodríguez-Gironés MÁ, Barbosa A (2013). Olfactory detection of dimethyl sulphide in a krill-eating Antarctic penguin. Mar Ecol Prog Ser.

[78] Hays GC, Åkesson S, Broderick AC, Glen F, Godley BJ, Papi F (2003). Island-finding ability of marine turtles. Proc R Soc Lond B Biol Sci.

[79] Girard C, Sudre J, Benhamou S, Roos D, Luschi P (2006). Homing in green turtles Chelonia mydas: oceanic currents act as a constraint rather than as an information source. Mar Ecol Prog Ser.

[80] Galli S, Gaspar P, Fossette S, Calmettes B, Hays GC, Lutjeharms JRE (2012). Orientation of migrating leatherback turtles in relation to ocean currents. Anim Behav.

[81] Putman NF, Mansfield KL (2015). Direct Evidence of Swimming Demonstrates Active Dispersal in the Sea Turtle “Lost Years”. Curr Biol.

[82] Kobayashi DR, Farman R, Polovina JJ, Parker DM, Rice M, Balazs GH (2014). “Going with the flow” or not: evidence of positive rheotaxis in oceanic juvenile loggerhead turtles (*Caretta caretta*) in the South Pacific Ocean using satellite tags and ocean circulation data. PLoS One.

[83] Lohmann KJ, Lohmann CMF, Endres CS (2008). The sensory ecology of ocean navigation. J Exp Biol.

[84] DeBose JL, Nevitt GA (2008). The use of odors at different spatial scales: comparing birds with fish. J Chem Ecol.

[85] Guilford T, Freeman R, Boyle D, Dean B, Kirk H, Phillips R (2011). A dispersive migration in the Atlantic puffin and its implications for migratory navigation. PLoS One.

[86] Clucas GV, Younger JL, Kao D, Rogers AD, Handley J, Miller GD (2016). Dispersal in the sub-Antarctic: king penguins show remarkably little population genetic differentiation across their range. BMC Evol Biol.

[87] Cristofari R, Trucchi E, Whittington JD, Vigetta S, Gachot-Neveu H, Stenseth NC (2015). Spatial heterogeneity as a genetic mixing mechanism in highly philopatric colonial seabirds. PLoS One.

