# Supplementary material for: Exploration during early life: distribution, habitat and orientation preferences in juvenile king penguins
Source: Mov Ecol. 2019 Oct 21;7:29. doi: 10.1186/s40462-019-0175-3 (PMC6805568; doi:10.1186/s40462-019-0175-3)
Supplement: Supplementary file 1 — Additional file 1. Supplementary Tables and Figures about individuals tracking parameters, models outputs, distribution maps, PCA of environmental variables during the winter, clustering outputs and time series. [file 40462_2019_175_MOESM1_ESM.docx]

**Exploration during early life: distribution, habitat and orientation preferences in juvenile king penguins**

**Orgeret F.*, Péron C., Enstipp M., Delord K., Weimerskirch H. & Bost C.A.**

***Corresponding author:** florianorgeret@gmail.com

**Supplementary Material**

**Table S1:** Summary of tracking parameters for juvenile king penguins (5 individuals which had the shortest monitoring duration and traveling distance are: #133688, #133692, #133695, #133700 and #141650).

| **Id** | **Start date** | **End date** | **Deployment duration (days)** | **Nr. of transmitted days** | **Nr. of daily location fixes** | **Foraging range  (km)** | **Total distance  (km)** | **Daily distance (km)** | **Max daily distance  (km)** |
| --- | --- | --- | --- | --- | --- | --- | --- | --- | --- |
| 133688 | 22/12/2013 | 07/03/2014 | 75.9 | 26 | 14.9±3.1 | 1004.2 | 3317.2 | 60.7±26.7 | 113.9 |
| 133689 | 19/12/2013 | 17/10/2014 | 302.7 | 83 | 12.3±7.1 | 4392.2 | 13258.8 | 54.2±23.9 | 126.5 |
| 133690 | 28/12/2013 | 30/09/2014 | 276.8 | 80 | 14.9±6.7 | 2986.0 | 12407.2 | 58.4±28.8 | 131.5 |
| 133691 | 19/12/2013 | 09/09/2014 | 264 | 79 | 14.3±4.6 | 3492.8 | 11374.7 | 54.4±28.3 | 185.2 |
| 133692 | 25/12/2013 | 10/03/2014 | 74.4 | 25 | 15.4±4.1 | 1811.2 | 3416.9 | 60.7±21.2 | 89.9 |
| 133693 | 28/11/2013 | 07/08/2014 | 252 | 68 | 14.6±4.8 | 3041.7 | 12952.4 | 68.1±32.1 | 189.8 |
| 133694 | 28/11/2013 | 15/09/2014 | 291.6 | 81 | 15.1±6.6 | 2060.3 | 11727.7 | 54.0±28.4 | 120.9 |
| 133695 | 19/12/2013 | 03/04/2014 | 105.1 | 33 | 15.9±5.9 | 1867.0 | 4356.6 | 50.7±20.1 | 86.0 |
| 133700 | 19/12/2013 | 14/03/2014 | 85 | 27 | 15.6±4.2 | 1632.8 | 4794.8 | 70.2±22.3 | 141.2 |
| 133701 | 19/12/2013 | 03/10/2014 | 288.8 | 86 | 16.5±6.8 | 3156.1 | 12197.1 | 55.9±29.6 | 137.1 |
| 133702 | 25/12/2013 | 24/09/2014 | 272.3 | 79 | 17.0±5.5 | 4654.6 | 12163.0 | 56.0±25.7 | 117.6 |
| 141650 | 10/12/2014 | 31/03/2015 | 111.8 | 36 | 14.5±5.6 | 2786.9 | 4941.6 | 55.5±34.1 | 129.4 |
| 141651 | 10/12/2014 | 13/08/2015 | 246.7 | 79 | 12.5±6.5 | 3509.2 | 10696.3 | 48.5±32.6 | 123.2 |
| 141652 | 18/01/2015 | 19/08/2015 | 213.7 | 66 | 14.5±9.7 | 3854.6 | 10847.0 | 49.8±32.2 | 113.5 |
| 141653 | 07/12/2014 | 29/06/2015 | 204.7 | 63 | 15.7±5.7 | 3868.7 | 10575.8 | 64.6±30.3 | 130.9 |
| 141654 | 10/12/2014 | 17/07/2015 | 219.7 | 68 | 14.5±6.9 | 3610.4 | 11441.7 | 58.9±28.9 | 113.9 |
| 141657 | 07/12/2014 | 11/07/2015 | 216.7 | 61 | 12.7±10.1 | 3969.2 | 11602.5 | 59.2±38.2 | 157.4 |
|  |  | *MEAN*±*SD* | *206.0*±82.3 | *61.2*±22.4 | *14.7*±*6.1* | *3041.0*±*1444.3* | *9533.6*±*3661.6* | *57.6*±*28.5* | *129.9*±*27.6* |

**Table S2:** Summary of tracking parameters for non-breeding adult king penguins

| **Id** | **Start date** | **End date** | **Deployment duration (days)** | **Nr. of transmitted days** | **Nr. of daily location fixes** | **Foraging range  (km)** | **Total distance  (km)** | **Daily distance (km)** | **Max daily distance  (km)** |
| --- | --- | --- | --- | --- | --- | --- | --- | --- | --- |
| 133697 | 13/03/2014 | 20/10/2014 | 221.1 | 81 | 11.8±9.3 | 2692.6 | 10369.5 | 38.3 | 118.7 |
| 133699 | 08/03/2014 | 04/08/2014 | 148.8 | 124 | 16.4±5.1 | 2660.9 | 9315.7 | 63.2 | 153.6 |
| 141649 | 20/02/2015 | 12/10/2015 | 234.1 | 53 | 14.8±14.7 | 1874.1 | 8929.1 | 55.7 | 117.0 |
| 141655 | 23/02/2015 | 23/10/2015 | 242.2 | 68 | 13.9±8.0 | 1670.6 | 11053.3 | 55.4 | 166.0 |
| 141656 | 21/02/2015 | 26/10/2015 | 246.4 | 65 | 15.4±7.7 | 1957.3 | 11664.7 | 58.3 | 120.5 |
| 141658 | 18/02/2015 | 06/10/2015 | 230.2 | 49 | 17.0±10.2 | 1461.4 | 8487.6 | 60.3 | 138.3 |
|  |  | *MEAN*±*SD* | *220.5*±*36.2* | *73.3*±*27.3* | *14.9*±*9.2* | *2052.8*±513.0 | *9970.0*±1258.1 | *55.2*±*8.8* | *135.7*±*20.6* |

**Table S3:** Model outputs concerning habitat modeling/environmental features (best GAMM model; deviance explained = 23.1%). Significance is indicated by stars with ***<0.001 and **<0.01; ns=non-significant; edf: estimated degrees of freedom

|  | term | estimate | std.error | Z | p.value | signif |
| --- | --- | --- | --- | --- | --- | --- |
| parametric  coefficients | intercept | 1.54 | 0.18 | 8.35 | 0.00 | *** |
|  | year2014/15 | -0.46 | 0.28 | -1.67 | 0.09 | Ns |
|  | adults | 2.06 | 2.73 | 0.75 | 0.45 | Ns |
|  | autumn | -0.88 | 0.10 | -8.87 | 0.00 | *** |
|  | winter | 0.92 | 0.15 | 5.99 | 0.00 | *** |

|  | term | Edf | Chi^2^ | p.value | signif |
| --- | --- | --- | --- | --- | --- |
| smoothed  terms | s(mld):statutjuvs | 3.37 | 50.19 | 0.00 | *** |
|  | s(mld):statutnb | 0.98 | 2.22 | 0.11 | Ns |
|  | s(sst):statutjuvs | 6.56 | 174.38 | 0.00 | *** |
|  | s(sst):statutnb | 5.22 | 41.49 | 0.00 | *** |
|  | s(csp):statutjuvs | 2.49 | 17.57 | 0.00 | *** |
|  | s(csp):statutnb | 2.86 | 24.01 | 0.00 | *** |
|  | s(log(chla)):statutjuvs | 4.89 | 37.70 | 0.00 | *** |
|  | s(log(chla)):statutnb | 5.77 | 76.88 | 0.00 | *** |
|  | s(speed):statutjuvs | 4.27 | 76.28 | 0.00 | *** |
|  | s(speed):statutnb | 2.60 | 22.87 | 0.00 | *** |
|  | s(mlon.mlat):statutjuvs | 28.25 | 1127.73 | 0.00 | *** |
|  | s(mlon.mlat):statutnb | 20.01 | 471.40 | 0.00 | *** |
|  | s(ids) | 19.00 | 379.91 | 0.00 | *** |

**Table S4:** Model outputs concerning upwind orientation probability vs. Chlorophyll a concentration (best GAMM model; deviance explained = 14%). Significance is indicated by stars with ***<0.001 and **<0.01; ns=non-significant; edf: estimated degrees of freedom

|  | Term | estimate | | std.error | | Z | | p.value | | Signif | |
| --- | --- | --- | --- | --- | --- | --- | --- | --- | --- | --- | --- |
| Parametric  coefficients | Intercept | 0.77 | | 0.16 | | 4.70 | | 0.000 | | *** | |
|  | Adults | -0.48 | | 0.59 | | -0.83 | | 0.409 | | Ns | |
|  | Autumn | -0.33 | | 0.21 | | -1.58 | | 0.113 | | Ns | |
|  | Winter | -1.03 | | 0.25 | | -4.06 | | 0.000 | | *** | |
|  |  |  | |  | |  | |  | |  | |
|  | | Term | | Edf | | Chi^2^ | | p.value | | Signif | |
| Smoothed  terms | | s(log(chla1)):statutjuvs | | 2.62 | | 8.62 | | 0.018 | | * | |
|  |  | s(log(chla1)):statutnb | | 2.50 | | 15.66 | | 0.001 | | ** | |
|  |  | s(lon.lat):statutjuvs | | 26.38 | | 300.12 | | 0.000 | | *** | |
|  | | s(lon.lat):statutnb | | 9.85 | | 55.71 | | 0.000 | | *** | |
|  | | s(ids) | | 15.25 | | 61.01 | | 0.000 | | *** | |

**Table S5:** Model outputs concerning upstream orientation probability vs. Chlorophyll a concentration (best GAMM model; deviance explained = 6%). Significance is indicated by stars with ***<0.001 and **<0.01; ns=non-significant; edf: estimated degrees of freedom.

|  | Term | estimate | std.error | Z | p.value | Signif |
| --- | --- | --- | --- | --- | --- | --- |
| Parametric  coefficients | Intercept | 0.14 | 0.11 | 1.31 | 0.192 | Ns |
|  | Adults | -0.15 | 0.61 | -0.24 | 0.813 | Ns |
|  | Autumn | 0.15 | 0.13 | 1.16 | 0.247 | Ns |
|  | Winter | -0.38 | 0.16 | -2.36 | 0.018 | * |

|  | Term | Edf | Chi^2^ | p.value | Signif |
| --- | --- | --- | --- | --- | --- |
| Smoothed  terms | s(log(chla1)):statutjuvs | 3.32 | 13.08 | 0.008 | ** |
|  | s(log(chla1)):statutnb | 3.12 | 40.08 | 0.000 | *** |
|  | s(lon.lat):statutjuvs | 26.69 | 252.23 | 0.000 | *** |
|  | s(lon.lat):statutnb | 12.97 | 75.24 | 0.000 | *** |
|  | s(ids) | 15.77 | 80.12 | 0.000 | *** |


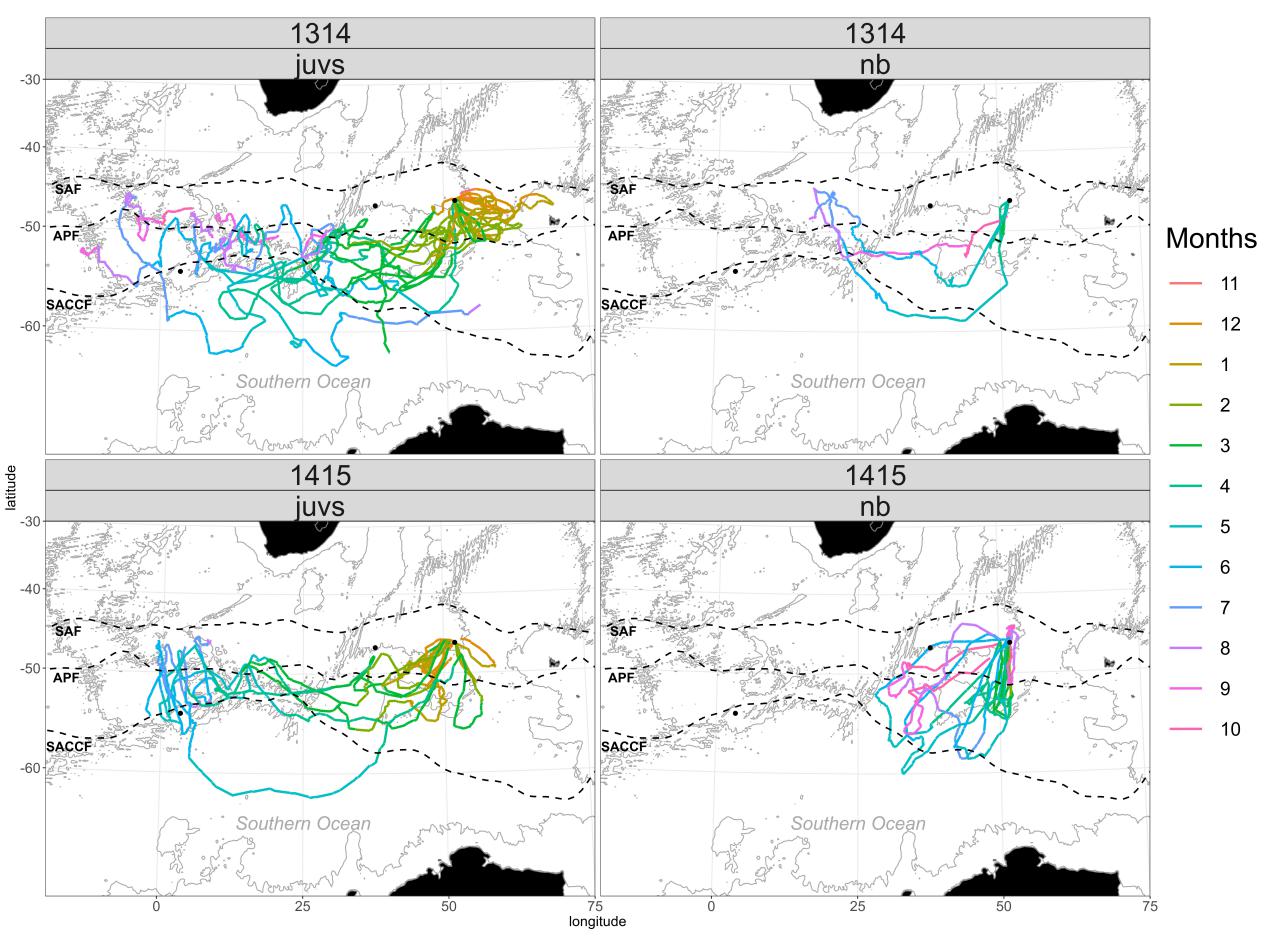


**Figure S1:** Movement tracks for all individuals across seasons, for both study years: juveniles (top/bottom left; N=11 for 2013/2014 and N=6 for 2014/2015) and non-breeding adults (top/bottom right; N=2 for 2013/2014 and N=4 for 2014/2015). See Figure 1 for the description and definitions of frontal lines. The grey contour lines in the background indicate the 2000 m depth isobath.


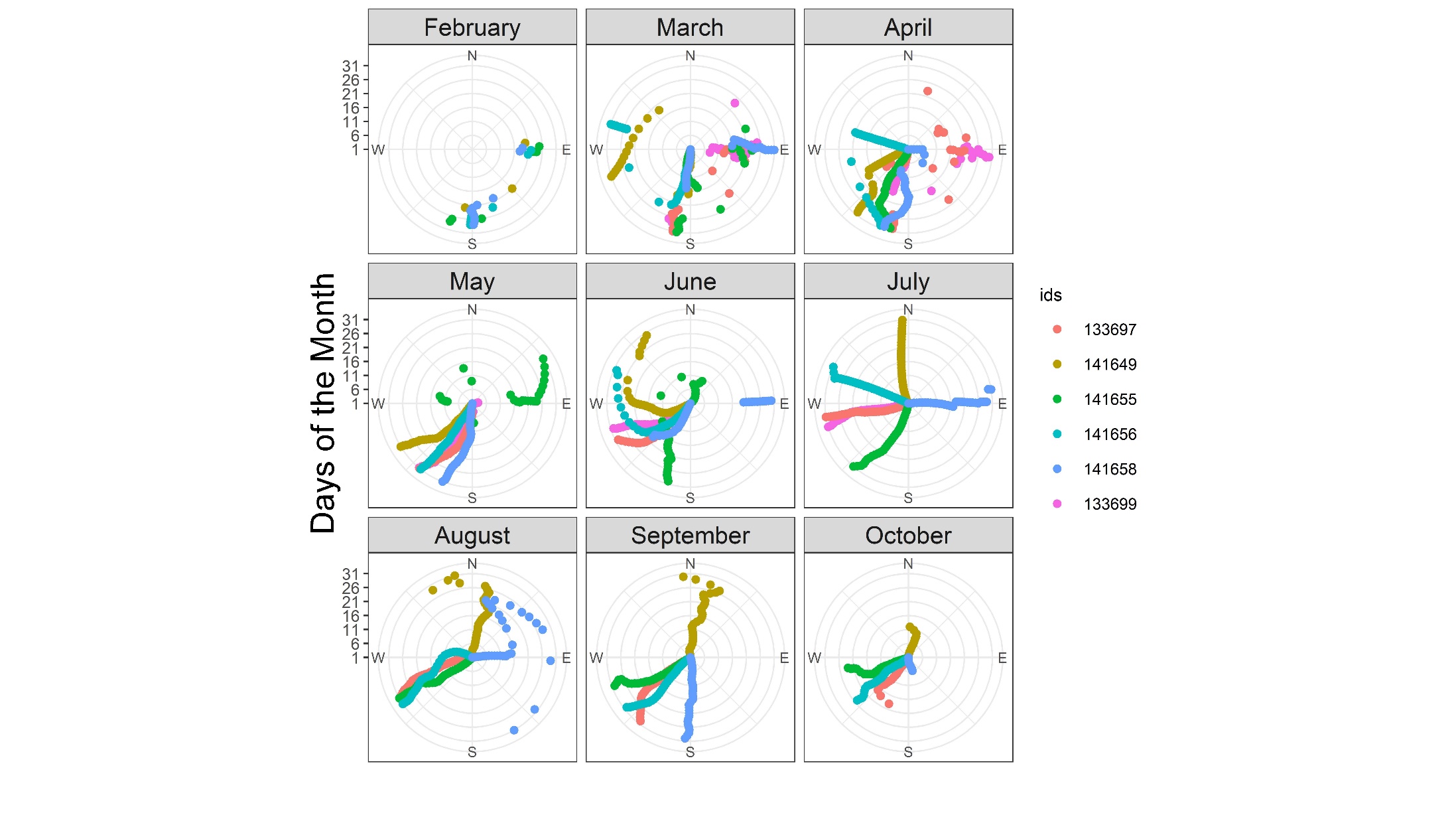


**Figure S2:** Daily directional positions of 6 non-breeding adult king penguins (color-coded) relative to their departure colony (innermost circle). Daily averages compass bearings are presented for each month, starting with adult departure in February until October of the following year. Within a monthly circle, time progresses from the inside out, with the outermost ring representing the end of the month. Data for both years were pooled.


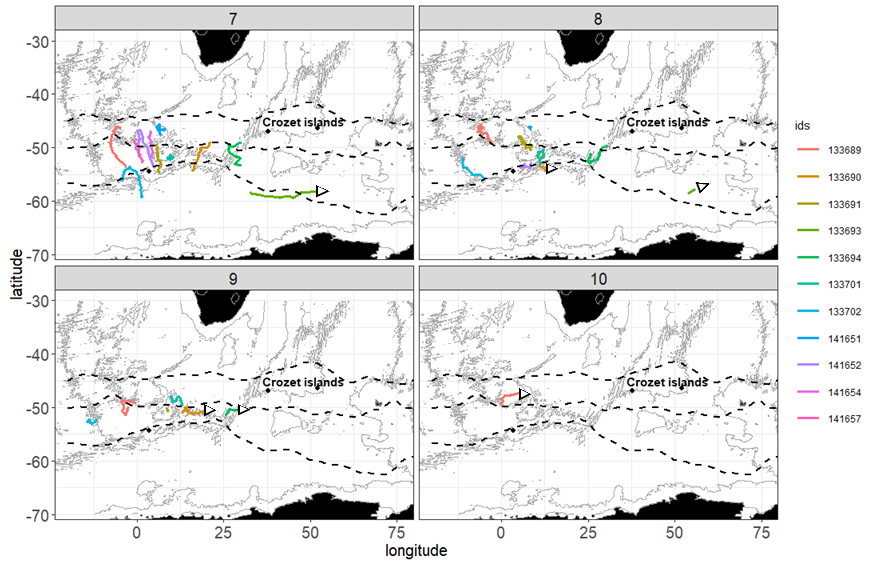


**Figure S3:** Juvenile tracks (N=11) illustrating the return phase during winter/spring. Numbers on top indicate months, while arrows indicate the start of the return towards the natal colony. The grey contour lines in the background indicate the 2000 m depth isobath.


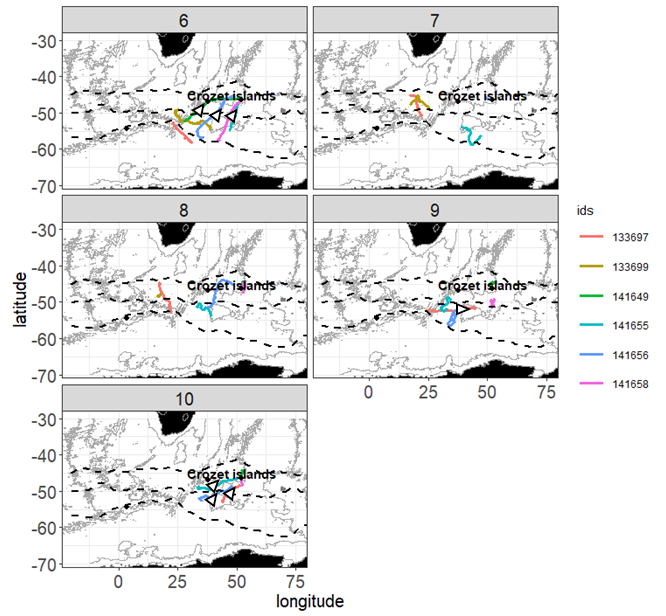


**Figure S4:** Tracks from adult non-breeders (N=6) illustrating the return phase towards the colony. Numbers on top indicate months, while arrows indicate the start of the return towards the colony. The grey contour lines in the background indicate the 2000 m depth isobath.


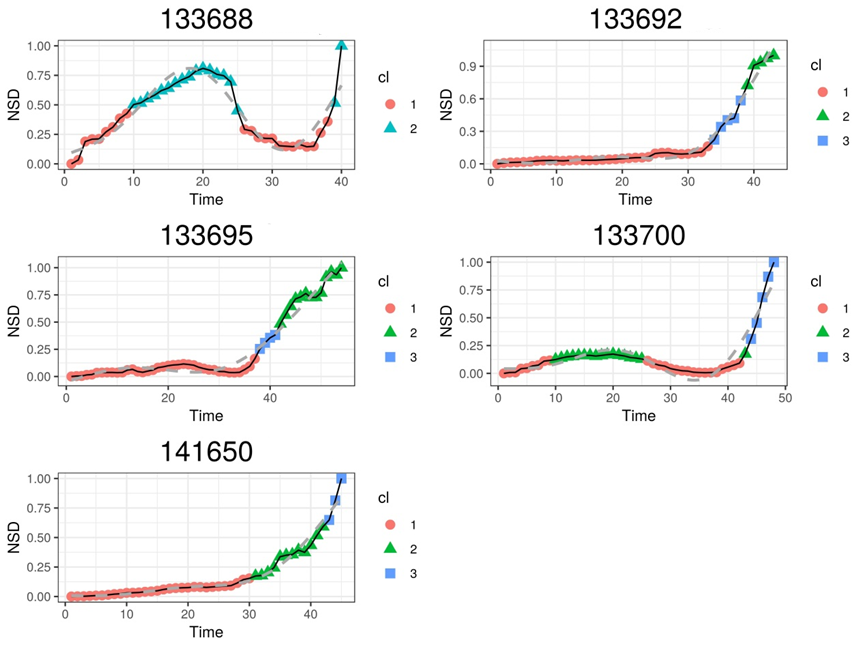


**Figure S5:** Output of Net Squared Displacement (NSD) models for 5 juveniles which had the shortest monitoring duration (Table S1) due to tags failure or death of birds. The colors and symbols (cl: 1-3) indicate the different latent states of juveniles and time indicates the number of tag transmission days since birds departed from the colony.


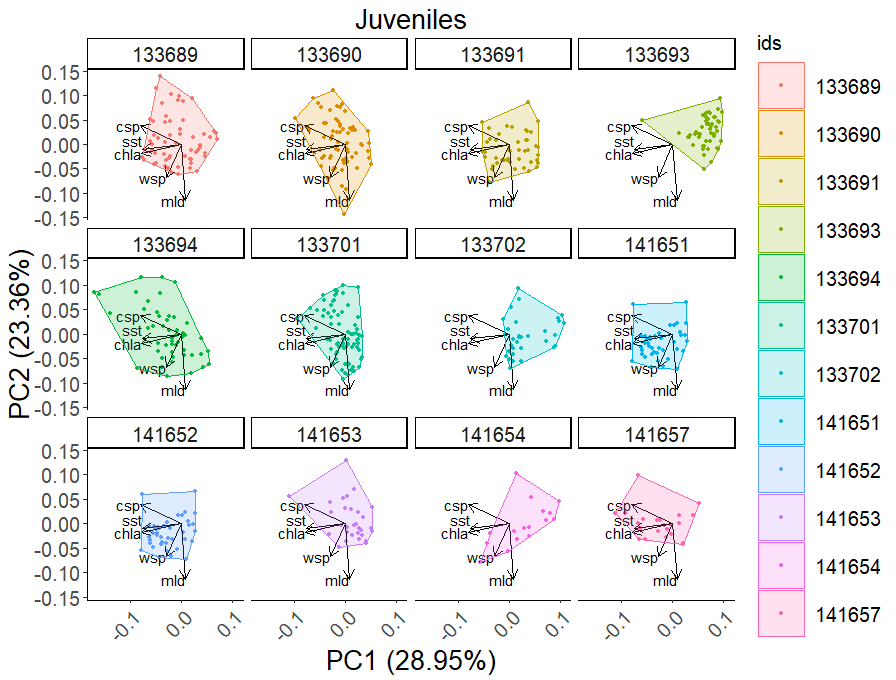


**Figure S6:** Principal Component Analysis for the daily environmental variables encountered by juveniles during ‘state 2’ (the final phase) of the latent state model (N=12). Note that juveniles that died during the first 3-4 months at sea (N=5) were not included in this analysis. csp: current speed; sst: sea surface temperature; chla: chlorophyll a; wsp: wind speed; mld: mixed layer depth.


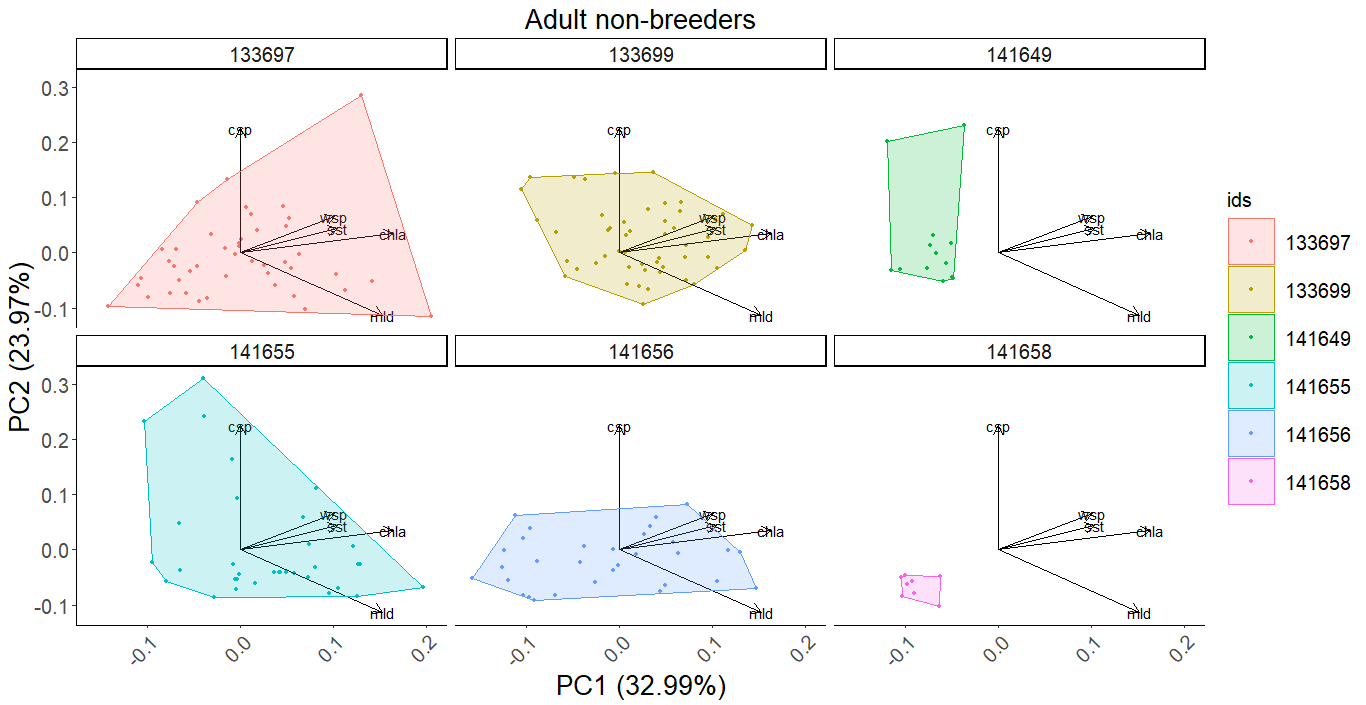


**Figure S7:** Principal Component Analysis for the daily environmental variables encountered by the adult non-breeders during ‘state 2’ (the final phase) of the latent state model (N=6). csp: current speed; sst: sea surface temperature; chla: chlorophyll a; wsp: wind speed; mld: mixed layer depth.


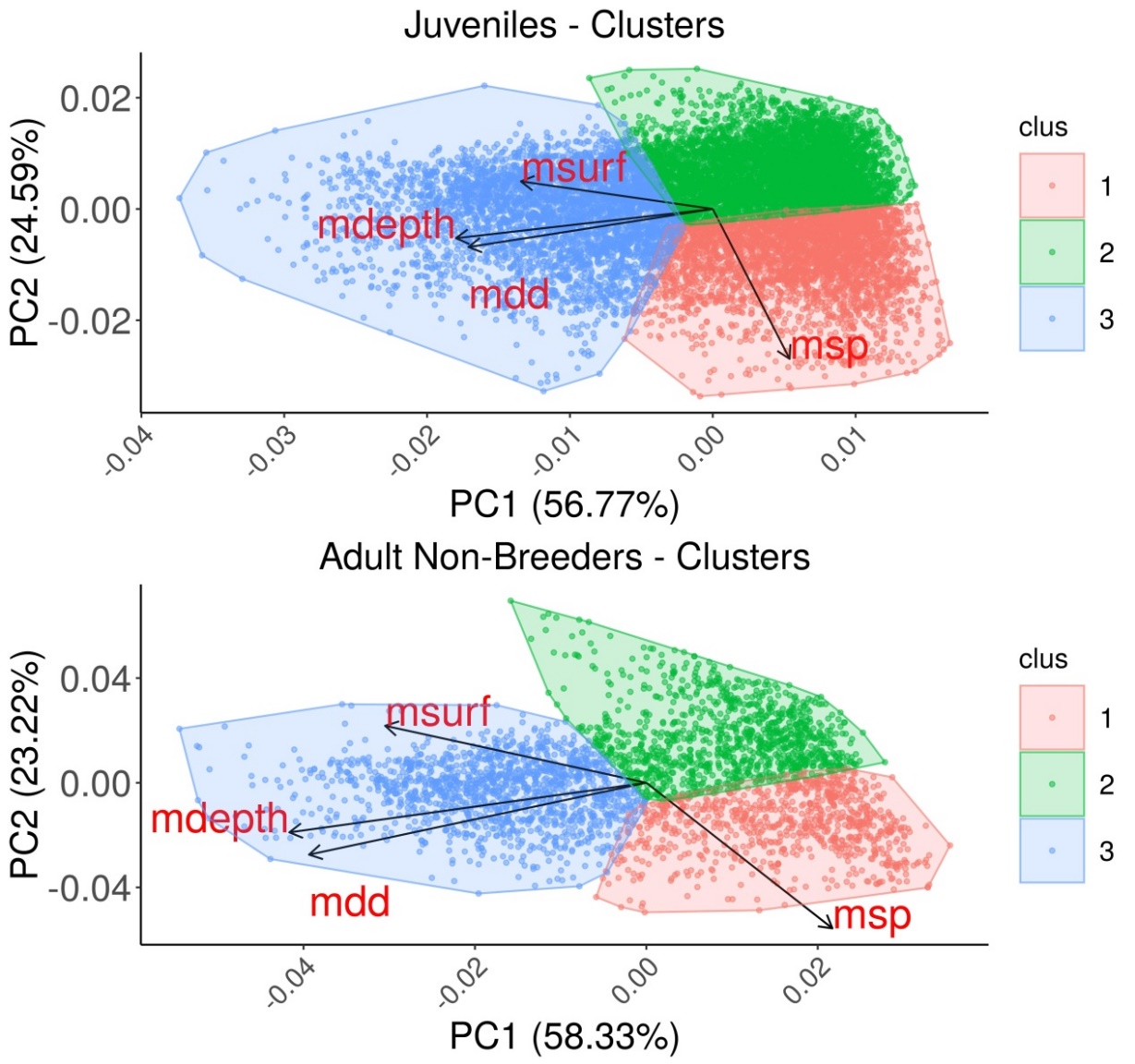


**Figure S8:** Principal Component Analysis used by the cluster algorithm allowed to distinguish between three clusters, according to hourly travel speed (msp) and hourly means for various dive parameters (mean maximum dive depth: mdepth; mean dive duration: mdd; and mean surface interval duration: msurf). Three behavioral modes could be distinguished (Cluster 1=traveling; 2/3=foraging). Results for juveniles (top) and non-breeding adults (bottom) are shown.


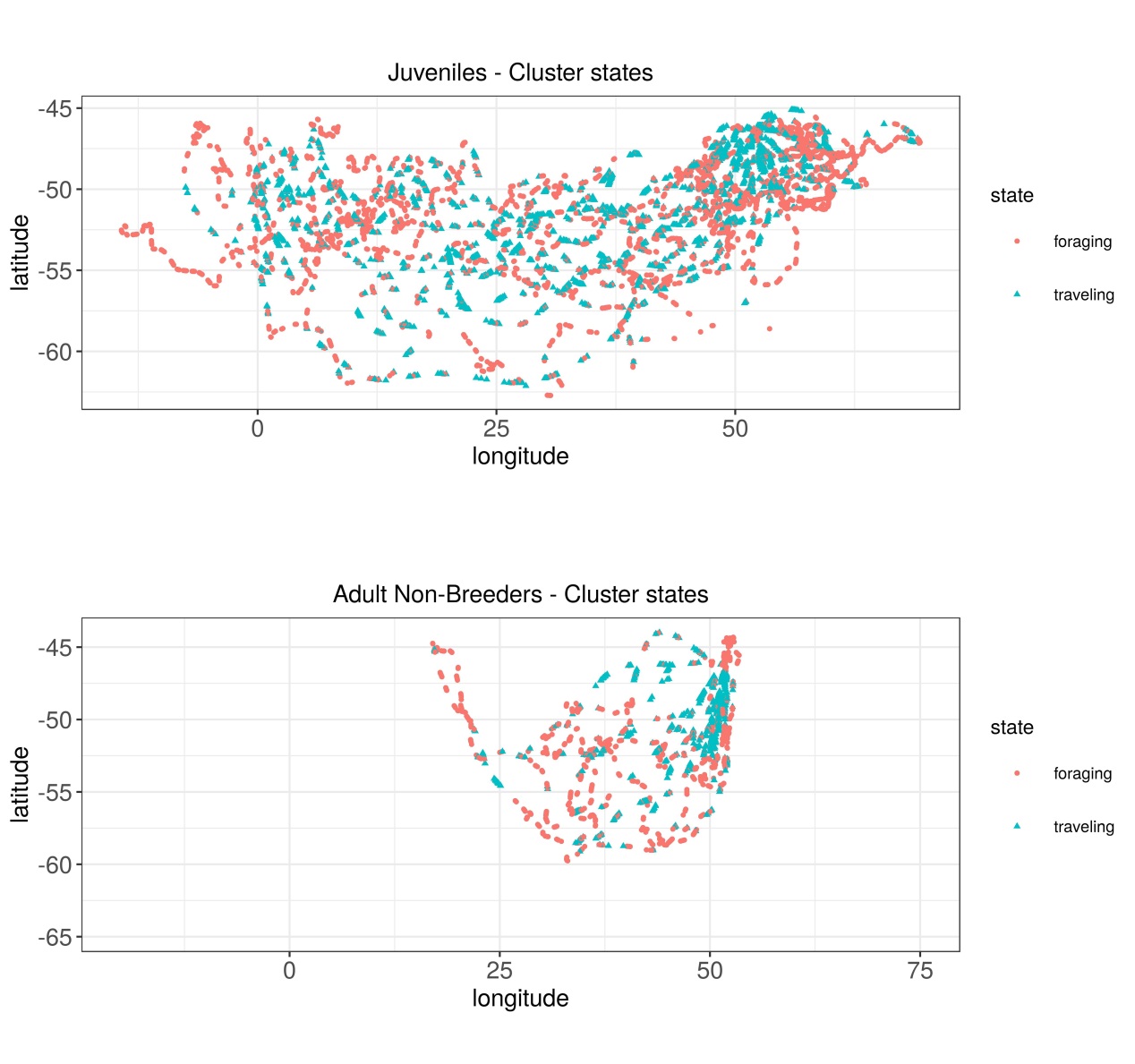


**Figure S9:** Locations for juvenile (top) and non-breeding adult king penguins (bottom) and associated dive modes (traveling/foraging), according to cluster analysis.


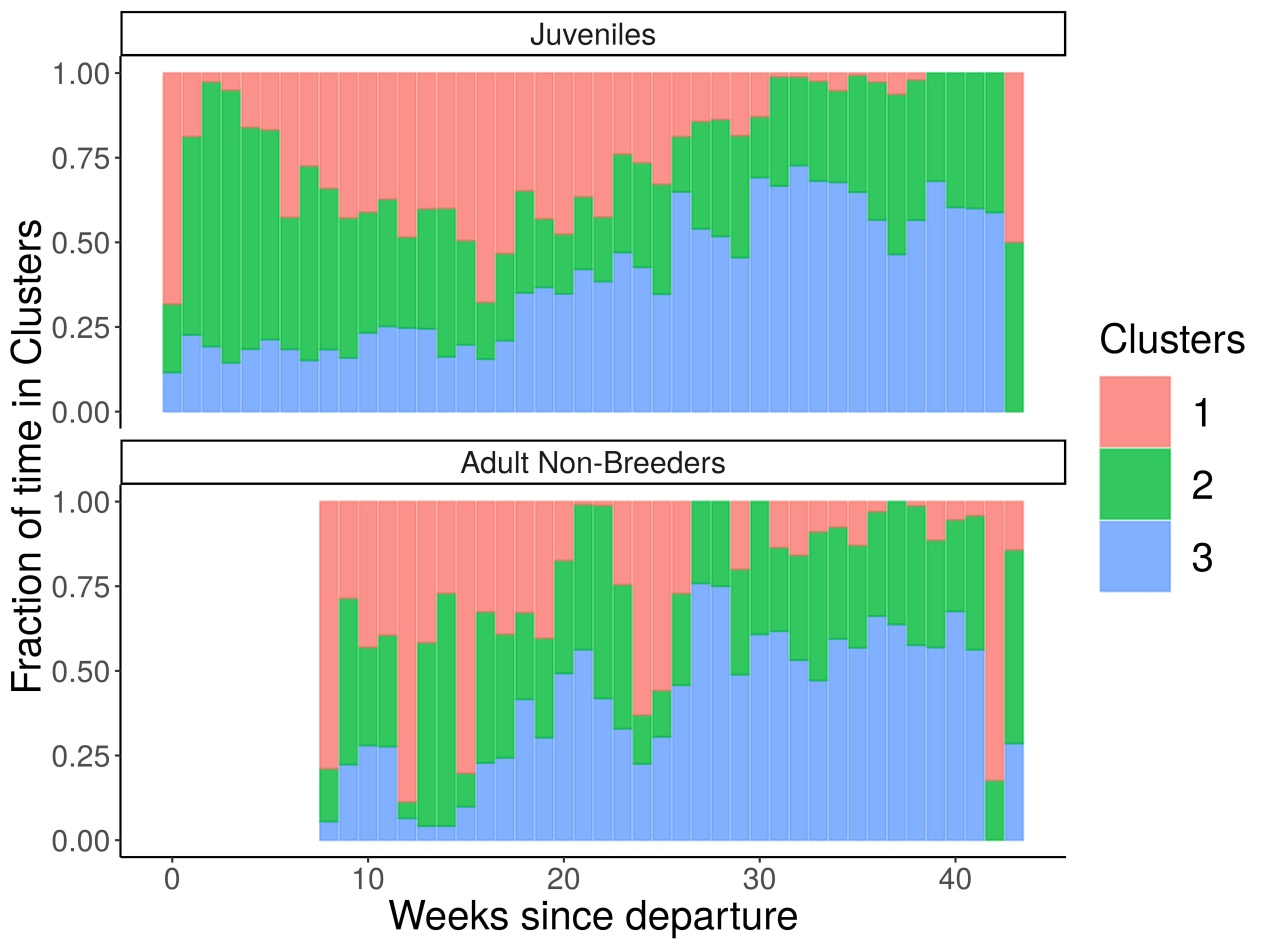


Figure S10: Relative time (weekly averages) that juveniles (top) and adult non-breeders (bottom) spent in different behavioral modes (1=traveling; 2/3=foraging), according to cluster analysis.
